# International practices, barriers, and enablers for patient and public involvement in pricing and reimbursement decision-making: a mixed-methods study

**DOI:** 10.3389/fphar.2026.1791567

**Published:** 2026-04-21

**Authors:** Alice Vanneste, Morgane Deknudt, Elise Schoefs, Charlotte Verbeke, Peter Sinnaeve, Diane-Estelle Ngatchou-Djomo, Jana Hlaváčová, Isabelle Huys, Tom Adriaenssens

**Affiliations:** 1 Department of Pharmaceutical and Pharmacological Sciences, KU Leuven, Leuven, Belgium; 2 Department of Cardiovascular Diseases, University Hospitals Leuven, Leuven, Belgium; 3 Department of Cardiovascular Medicine, KU Leuven, Leuven, Belgium; 4 Belgian umbrella patient organisation, Ligue des Usagers des Services de Santé (LUSS), Namur, Belgium; 5 Department of Health Economics, United Nations University – Maastricht Economic and Social Research Institute on Innovation and Technology, Maastricht, Netherlands; 6 International Relations and EU Unit, Ministry of Health of the Czech Republic, Prague, Czechia

**Keywords:** consultation, focus group discussions, international practices, participation, patient and public involvement, pricing and reimbursement, scoping literature review, semi-structured interviews

## Abstract

**Introduction:**

Patient and public involvement (PPI) in pricing and reimbursement (P&R) procedures is increasingly recognized as essential for strengthening the quality, legitimacy, and patient-centredness of healthcare decision-making. However, implementation remains heterogeneous across countries and data on concrete practices, barriers, and enablers are fragmented. This study aimed to map international approaches to PPI in P&R procedures and identify barriers and enablers to meaningful involvement.

**Methods:**

A mixed-methods design was applied, combining a scoping literature review complemented by qualitative research. The scoping literature review was conducted following PRISMA-ScR guidance to identify scientific publications on PPI in P&R procedures. Semi-structured interviews and focus group discussions were conducted with industry experts, policymakers, assessors, and patient organisations. A descriptive, narrative, and thematic analysis integrating findings across data sources identified recurring patterns, cross-country similarities and differences, and diverse stakeholders’ perspectives.

**Results:**

The literature and stakeholder perspectives revealed that PPI remains uneven and inconsistently implemented in P&R procedures across healthcare systems, though overall trends point towards more structured participation and partnership. Barriers include: (i) resource constraints, (ii) unclear value and impact of PPI, (iii) conflict of interest and confidentiality concerns, (iv) recruitment challenges, (v) concerns about representativeness and diversity, (vi) complexity of P&R terminology and processes, (vii) methodological gaps, (viii) limited experience with PPI, and (ix) competing workloads. Across countries, stakeholders identified a shared set of enabling conditions necessary for meaningful involvement: (i) clear institutional commitment and leadership, (ii) dedicated resources, time, and experience-building with explicit definition of roles, expectations, and scope of influence, (iii) transparency and systematic feedback for two-way communication, (iv) methodological rigour and evaluation frameworks for capturing and reporting experiential evidence, (v) inclusivity and representativeness with diverse participation opportunities, and (vi) capacity building for both patients and the public, as well as P&R agencies.

**Conclusion:**

While PPI in P&R procedures remains uneven across countries, there is a clear shift towards more structured participation. Meaningful and sustainable involvement requires the integration of structural, organisational, procedural, technical, and financial enablers. When these enablers are aligned and mutually reinforcing, PPI can become a substantive driver of more patient-centred, transparent, and socially accountable P&R decision-making.

## 1 Introduction

Across healthcare systems worldwide, there is growing recognition that patients and the wider public hold experiential knowledge that can strengthen the quality, legitimacy, relevance, and societal acceptability of health policy decision-making (Price et al., 2018; Arumugam et al., 2023). Their lived experiences provide essential context that clinical and economic evidence cannot fully capture (ICH, 2021). By incorporating these complementary insights, decision-makers can more accurately assess the real-world impact of health technologies, including treatment burden, side effects, functional impact, and broader quality-of-life and social considerations (Rand et al., 2019).

In recent decades, patient and public involvement (PPI) has become increasingly embedded in research, clinical guideline development, regulatory evaluation, and pricing and reimbursement (P&R) decision-making, including health technology assessment (HTA) (Whitty, 2013). PPI is commonly defined as decision-making done *with* or *by* patients or the public, rather than to, about, or for them, reflecting the foundational ‘Nothing about us without us’ principle of Charlton (Turk et al., 2017; Charlton, 1998). Evidence consistently demonstrates that PPI leads to more relevant research questions, improved clinical recruitment and participation, more informed benefit-risk evaluations, and reduced research waste by focusing on meaningful treatment outcomes that align with patients’ needs and preferences (Arumugam et al., 2023; Marzban et al., 2022; Skovlund et al., 2020; Crocker et al., 2018; Brett et al., 2014).

Despite growing momentum and regulatory agencies such as the European Medicines Agency (EMA) and US Food and Drug Administration (FDA) providing more direction towards participatory governance and patient-centred approaches (EMA, 2025; FDA, 2025), the implementation of PPI in P&R procedures remains heterogenous across Europe and internationally (Pickaert, 2025). The slow and cautious uptake reflects the complexity of PPI implementation and the persistence of multiple, interrelated barriers, including misconceptions about the scientific validity of patient-generated evidence and concerns that patients may introduce subjective or advocacy-driven bias (Single et al., 2019). As a result, many countries rely on unstructured, *ad hoc*, or informal approaches for PPI with practices ranging from no involvement to informing, consulting, and participation in select phases of P&R procedures (Puebla et al., 2025; Wale et al., 2021). Without clear structures, defined roles, and methodological guidance, decision-makers may lack patient-relevant insights necessary to guide effective and meaningful P&R decision-making. These decisions ultimately determine patient access to medicines and shape resource allocation (Wale et al., 2021).

As international interest grows, countries are increasingly considering whether and how to strengthen or formalise PPI approaches within their P&R procedures. By understanding and learning from other agencies which PPI practices work, how they work, and under what circumstances, they can adapt processes to their own institutional context (Silva et al., 2021). However, published evidence on international practices and assessments of PPI activities and their influence on decision-making remains scarce and fragmented, with only a small number indicating specific constraints and opportunities unique to PPI in P&R decisions (Single et al., 2019; Dipankui et al., 2015; Weeks et al., 2017). Moreover, empirical data capturing the perspectives of policymakers, assessors, industry experts, and patient organisations on PPI implementation remains limited, leaving decision-makers without clear evidence on the effectiveness, added value, or practical requirements for PPI to guide policy development (Holtorf et al., 2024). Therefore, a comprehensive, practice-informed assessment of current international practices and stakeholders’ perspectives is urgently needed.

This study addresses this gap by examining international PPI practices and the barriers and enablers for involving patients and the public in P&R decision-making. By combining insights from scientific literature with qualitative stakeholder interviews and focus group discussions, this research identifies the structural, organisational, procedural, financial, and technical determinants that shape PPI in P&R procedures. The findings aim to offer a comprehensive overview of current practices including barriers and enablers to inform decision-making for policymakers, P&R agencies, and patient communities seeking to design sustainable and meaningful PPI frameworks that enhance transparency, accountability, and patient-centred P&R decision-making.

## 2 Methods

This study adopted a mixed-methods design, in which a scoping literature review was conducted to map existing evidence, followed by qualitative data collection to explore stakeholder experiences in real-world practice. Findings from both components were integrated during analysis and synthesis. This sequential design was chosen to integrate the breadth of evidence for the literature with in-depth insights from stakeholder perspectives, providing a comprehensive and complementary understanding of PPI in P&R procedures.

### 2.1 Scoping literature review

A scoping review was conducted to map current PPI processes in P&R procedures across various countries, including barriers, enablers, and best practices. The review followed the PRISMA-ScR guidelines. The study protocol was registered on the Open Science framework (Vanneste et al., 2025) prior to data extraction.

A comprehensive search strategy was developed in collaboration with a trained medical librarian and researchers from KU Leuven. Peer-reviewed literature was searched on 17 December 2024 across three databases: PubMed (including Medline), Embase, and Scopus. The search combined two primary concepts: (i) patient and public involvement and (ii) pricing and reimbursement, with relevant synonyms, MeSH terms (for PubMed), and Emtree terms (for Embase) (see full search string in Supplementary Material 1). Retrieved articles were de-duplicated in EndNote and Rayyan.

A 10% pilot title-and-abstract screening was conducted in Rayyan double-blind by either AV or ES and MD to test and refine the inclusion criteria, being active involvement of patients or the public in P&R procedures focusing on practical and organisational aspects, barriers, enablers, best practices, or recommendations (Supplementary Material 1). Discrepancies were discussed and resolved in a consensus meeting with the research team (AV, MD, ES, and CV). Following the pilot phase, all remaining titles and abstracts were screened double-blind by AV or ES and MD. Full-text screening was then conducted independently by AV and MD. In cases of disagreement, a third researcher (ES) was consulted to reach consensus on final inclusion.

Following the screening, a Microsoft Excel data extraction form (Supplementary Material 1) was developed collectively by the research team. Extracted variables included descriptive information (e.g., title, author, publication year, country) and content parameters (e.g., type of PPI process, stakeholders involved, best practices, reported challenges and enablers). The extraction form was iteratively refined and independently piloted by two researchers (AV and MD) on a sample of included studies (n = 7; 10%). After ensuring clarity and consistency, one reviewer (AV) completed the full data extraction.

The analysis was guided by the initial data extraction form based on existing literature on PPI in P&R procedures, while remaining open to additional concepts emerging from the included studies. Data were analysed using both descriptive summaries to characterise the included studies and narrative synthesis to summarise and integrate findings across studies by identifying recurring patterns, conceptual categories, and cross-country similarities and differences. Countries discussed in the literature were grouped into analytical clusters to facilitate cross-country comparison. The Mixed Methods Appraisal Tool (MMAT) was used as a critical appraisal tool for the empirical studies (quantitative, qualitative, and mixed-methods study designs) included in the scoping literature review.

### 2.2 Qualitative research

Qualitative research including semi-structured interviews and focus group discussions was conducted to obtain more in-depth insights into the practical aspects of PPI to complement and contextualise findings from the scoping review. Three main stakeholder groups were included: (i) pharmaceutical industry experts, (ii) policymakers (including assessors), and (iii) patient organisations (including umbrella organisations).

#### 2.2.1 Participant recruitment

For the semi-structured interviews, a purposive sampling strategy was applied to search for stakeholders across Europe with relevant experience in PPI within P&R procedures (Robinson and Michalos, 2014). Potential participants were identified through the research team’s internal networks at supra-national and national levels, targeted online searches, and professional networking platforms such as LinkedIn. Invited individuals were contacted via email including an information sheet and informed consent form. Those who agreed to participate signed and returned the informed consent form.

As part of a 1-day seminar, focus group discussion sessions were held with patient organisations. These sessions were part of a meeting organized by an external organization (BeLux Experience Exchange for Patient Organisations BEEPO by Roche), which was targeted at representatives of patient organisations in Belgium and Luxembourg. Participants were informed about the study purpose before the start of each session and consent for participation and audio-recording was obtained.

#### 2.2.2 Data collection

A semi-structured interview guide, developed based on the study objectives and insights from the literature review, was used to ensure consistency while allowing flexibility for exploration based on participants’ input. Interviews were conducted online between March and May 2025 via Microsoft Teams in Dutch, French, or English, depending on participant preference, and lasted approximately 1 hour.

The focus group discussion sessions used a semi-structured format aligned with the interview topic guide, but adapted for group interaction. Two 1-h sessions were performed in-person in June 2025 during the BEEPO meeting in Brussels.

#### 2.2.3 Data analysis

All interviews and sessions were recorded, transcribed ad verbatim in the original language, and pseudonymised to ensure participant confidentiality. Transcripts were analysed using the thematic framework approach, following the principles described by Lacey and Luff (Lacey and Luff, 2007). This approach enables systematic coding within a predefined framework while allowing for modifications based on new concepts emerging from the data. A pilot was conducted to assess the clarity, relevance, and usability of the preliminary framework developed by AV and MD. Based on this pilot, the coding structure was refined collaboratively within the research team to ensure consistency and identify any necessary adjustments. The revised framework was then applied to all transcripts, allowing for the identification and organisation of key themes related to barriers and enablers for PPI.

Coding was performed in NVivo by one researcher (MD) and reviewed by a second researcher (AV) for validation. Following coding, individual stakeholder statements and aggregated code summaries were extracted in Excel to facilitate comparison across stakeholder groups and themes. The synthesis and interpretation of themes were discussed within the research team. Statements reflect either individual stakeholder perspectives, identified by participant codes, or aggregated insights derived from patterns across interviews and focus group discussions.

Findings from the qualitative research were analysed in parallel with, and integrated into, the findings of the scoping review, allowing barriers and enablers identified in the literature to be compared, complemented, and deepened by stakeholders’ insights. This triangulation process enabled confirmation, refinement, and contextualisation of results. The integrated analysis provided a comprehensive understanding of recurring patterns, divergent perspectives, and overarching themes related to PPI in P&R procedures, reflecting both documented literature evidence and real-world stakeholder experiences and thereby strengthening the validity and comprehensiveness of the study.

## 3 Results

### 3.1 Characteristics of included data sources

**FIGURE 1:**
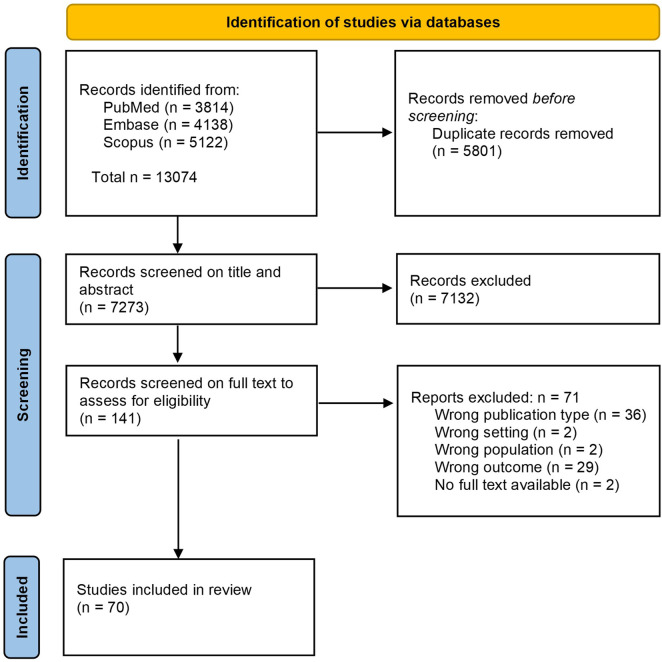
PRISMA flowchart of study identification and selection for the scoping literature review.

**FIGURE 2:**
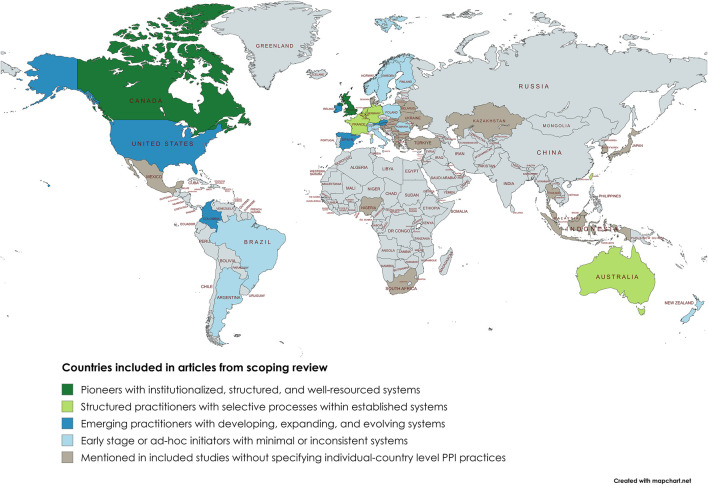
Geographic distribution of countries represented in the articles from the scoping review. The map displays all countries mentioned in the included studies from the scoping literature review, whether describing existing PPI practices in P&R procedures, ongoing developments, and emerging or planned initiatives. Countries were classified according to four clusters: (i) pioneers with institutionalised, structured, and well-resourced systems, (ii) structured practitioners with selective processes within established systems, (iii) emerging practitioners with developing, expanding, and evolving systems, and (iv) early stage or *ad hoc* initiators with minimal or inconsistent systems. Some countries could not be allocated to one of the clusters as they were mentioned in the studies (e.g., included in an international survey) but individual-country level PPI practices were not discussed. Consequently, these countries do not reflect the presence or absence of PPI practices.

For the scoping review, a total of 70 articles were included (Figure 1), published between 2006 and 2025. Most were primary studies (n = 56), followed by secondary research (n = 6) and mixed-design studies (n = 8). Among the primary studies, 20 used quantitative methods (e.g., surveys, document analyses), 17 qualitative methods (e.g., interviews, focus group discussions), 13 mixed-methods, and six other designs such as framework or protocol studies (Supplementary Material 2). Only three studies focused on a single disease area or technology. In total, 61 studies reported country-specific or cross-national PPI practices, while nine studies discussed conceptual or non-country specific models. Countries most frequently covered in the studies were Canada (n = 30), the United Kingdom (n = 18), Australia (n = 10), Germany (n = 10), the Netherlands (n = 10), and France (n = 8) (Figure 2). A more detailed summary of article characteristics is provided in Supplementary Material 3, as well as the MMAT checklist in Supplementary Material 4.

For the qualitative study, 14 participants were included in the semi-structured interviews, and the two focus group discussion sessions both included nine participants (Table 1). Participants were residents or represented organisations in a range of countries, including Austria, Belgium, the Czech Republic, France, Ireland, Luxembourg, Malta, and the United Kingdom. Participants operated at different geographical levels, including national, European, and global levels. Results from the qualitative study are integrated alongside the scoping review findings to provide an integrated mixed-methods perspective.

**TABLE 1:** Stakeholder groups included in the semi-structured interviews and focus group discussion sessions.

Stakeholder	Organisation type	Geographical scope	Qualitative study	Amount
Industry experts	Umbrella pharmaceutical organisation	National level	Interviews	2
	Pharmaceutical company	Global level	Interviews	2
Policymakers	HTA body	National level	Interviews	1
	P&R agency	National level	Interviews	1
	HTA and P&R agency	National level	Interviews	1
	Government authority	National level	Interviews	2
Patient organisations	Disease-specific patient organisation^a^	National and European level	Interviews + focus group	2 + 11
	Rare disease-specific patient organisation^a^	National level	Interviews + focus group	2 + 4
	Umbrella patient organisation	National level	Interview	1
	Patient support group	National level	Focus group	3

HTA, health technology assessment; P&R, pricing and reimbursement.

^a^ Examples of disease areas represented include breast cancer, lung cancer, Parkinson’s disease, haemophilia, COPD, multiple sclerosis, diabetic macular degeneration, diabetes, Crohn’s disease, Usher syndrome, Duchenne muscular dystrophy, and Huntington’s disease.

### 3.2 Conceptual and theoretical frameworks for PPI in P&R

The literature conceptualized PPI depending on its purpose, timing, and degree of influence. Several frameworks were identified. Gauvin’s model was most frequently mentioned, situating PPI at three domains, being policy making, organisational, and research (Menon and Stafinski, 2011; Gauvin et al., 2010; Gagnon et al., 2011; Lopes ACF. et al., 2020; Castro and Elias, 2018). Rowe and Frewer’s typology classified engagement into communication, consultation, and participation based on the information flow and decision-making power (Abelson et al., 2016; Wortley et al., 2016; Lopes et al., 2016; Facey et al., 2010). The IAP2 spectrum of public engagement (Wortley et al., 2016; Cleemput et al., 2015) and Arnstein’s ladder of citizen participation (Menon and Stafinski, 2011) further emphasize progressive empowerment from informing and consulting to involvement, collaboration, and empowerment with decision-making control. Abelson’s framework added a practical roadmap integrating principles, goals, and concepts with evaluation metrics (Dipankui et al., 2014; Häme et al., 2016; Kleme et al., 2014).

**FIGURE 3:**
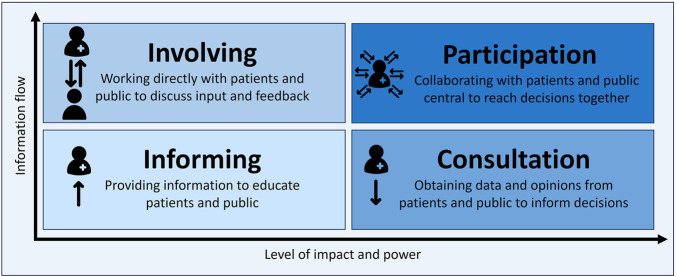
Levels of patient and public involvement based on information flow and level of impact and power.

In HTA-specific contexts, Gagnon’s model distinguishes involvement across different stages, including topic selection, evidence evaluation, and results dissemination (Gagnon et al., 2015; Atfeh et al., 2024), and Facey’s categories identified direct engagement and scientific evidence generation on patients’ values as two core dimensions (Castro and Elias, 2018; Facey et al., 2010). Other operational tools included the EUnetHTA core model, the HTA international (HTAi) interest group for patient and citizen involvement (PCIG) guidance, and national frameworks such as Spain’s RedETS framework (Toledo-Chávarri et al., 2020a; Stewart et al., 2021; Toledo-Chávarri et al., 2020b). Overall, these frameworks provide a conceptual basis for PPI defined across different levels of involvement based on information flow from one-way to two-way processes and level of impact and power in decision-making (Figure 3).

### 3.3 When to involve patients and the public across P&R procedures

The literature revealed that PPI can occur across P&R procedures, although its scope, format, and depth vary. During the earliest phases of HTA topic identification and prioritization, patients propose assessment requests or comment on relevance to unmet needs and evidence gaps (Menon and Stafinski, 2011; Castro and Elias, 2018; Abelson et al., 2016; Gagnon et al., 2015; Moran and Davidson, 2011; Gagnon et al., 2012). In the scoping phase, patients help define research questions, objectives, scope, and meaningful outcomes (Weeks et al., 2017; Menon and Stafinski, 2011; Abelson et al., 2016; Scott et al., 2017; Elvsaas et al., 2021; Lips et al., 2022; Gagnon et al., 2014a). At the evidence assessment stage, patients are consulted to complement literature-based evidence (Whitty, 2013; Gagnon et al., 2011; Abelson et al., 2016; Facey et al., 2010; Gagnon et al., 2015; Gagnon et al., 2012; Elvsaas et al., 2021). Typical methods include surveys, interviews, focus group discussions, weblog analyses, or structured patient submission templates capturing disease impact, treatment experiences, and expectations. The appraisal and recommendation phase mainly features direct participation, where patient or public representatives join deliberations, with or without vote, to present experiences, contextualize evidence, clarify uncertainties, and highlight social or ethical considerations (Whitty, 2013; Weeks et al., 2017; Menon and Stafinski, 2011; Abelson et al., 2016; Gagnon et al., 2015; Scott et al., 2017; Lips et al., 2022; Kreis and Schmidt, 2013; Hailey et al., 2013; Gunn et al., 2023). Their input shapes interpretation and framing of recommendations. Patient and public consultation provides an opportunity to comment on draft reports. Finally, at the dissemination stage, patient involvement informs the development of plain-language summaries of HTA reports and information materials to ensure accessibility (Weeks et al., 2017; Menon and Stafinski, 2011; Castro and Elias, 2018; Gagnon et al., 2015; Hailey et al., 2013).

While some systems involve patients and the public continuously, most concentrate engagement at specific stages, often with more PPI towards end stages (Abelson et al., 2016; Scott et al., 2017; Lips et al., 2022; Kreis and Schmidt, 2013). Direct participation is recognized as most valuable at the start and end of the HTA lifecycle, while consultation is best suited to evidence evaluation (Gagnon et al., 2014a). PPI was considered especially relevant for rare diseases, areas with limited clinical evidence, and technologies with major implications on quality of life, acceptability, feasibility, or ethical issues (Gagnon et al., 2014a; Hashem et al., 2018; Lopes E. et al., 2020; Tran et al., 2024).

### 3.4 Levels and forms of involvement across countries

Across countries, PPI was operationalized along a continuum from informing and communicating to consultation and active participation, depending on institutionalisation, decision-making influence, and resourcing. Four clusters emerged from the included literature in the scoping review: (i) pioneers with institutionalised, structured, and well-resourced systems, (ii) structured practitioners with selective processes within established systems, (iii) emerging practitioners with developing, expanding, and evolving systems, and (iv) early-stage or *ad hoc* initiators with minimal or inconsistent systems.

#### 3.4.1 Pioneers: institutionalised, structured, and well-resourced systems

The United Kingdom and Canada represent the most advanced and comprehensive systems. Both have formalized patient participation early on and have continuously expanded and optimized their approaches, establishing themselves as pioneers before other countries adopted similar practices later.

The United Kingdom has been a global pioneer with parallel but distinct processes. At the National Institute for Health and Care Excellence (NICE) (Weeks et al., 2017; Menon and Stafinski, 2011; Gagnon et al., 2011; Castro and Elias, 2018; Moran and Davidson, 2011; Scott et al., 2017; Kreis and Schmidt, 2013; Hashem et al., 2018; Young et al., 2017; Messina and Grainger, 2012; Livingstone et al., 2021; Johnson et al., 2024), the HTA body that directly informs reimbursement decisions, PPI is supported by a dedicated Public Involvement Programme (PIP). It offers continuous training and standardised templates such as the Summary of Information for Patients (SIP). A PIP adviser supports and develops involvement practices for both patients and the public in different formats. Patient organisations contribute during scoping workshops, submit written evidence via NICE’s PIP template, and nominate up to two patient experts to attend appraisal committee meetings to support the written submission, clarify issues, and participate in discussions. Two voting lay members with an understanding of the patient and public perspective are also included. Members of the public can comment on draft guidance and observe the committee meetings. Since 2002, the Citizen Council, consisting of public members reflective of the adult United Kingdom population, has provided a societal perspective on value and ethical issues (Kreis and Schmidt, 2013; Messina and Grainger, 2012). Scotland’s (Weeks et al., 2017; Menon and Stafinski, 2011; Stewart et al., 2021; Scott et al., 2017; Young et al., 2017; Na et al., 2023; Cook et al., 2021) Scottish Medicines Consortium (SMC) operates a comparable model through its Public Involvement Network (PIN), which supports and trains patient groups, evaluates and develops PPI processes, includes citizen members in HTA committees, and requires SIP patient submissions in industry HTA to ensure clarity of the assessed technology. Wales (Scott et al., 2017; Young et al., 2017; Na et al., 2023) similarly maintains a Patient and Public Involvement Standing Group to support engagement in its HTA processes.

In Canada (Menon and Stafinski, 2011; Scott et al., 2017; Messina and Grainger, 2012; Na et al., 2023; Gagnon et al., 2021; Bidonde et al., 2021; Berglas et al., 2016; Rocchi et al., 2015; Poder et al., 2020; Mercer et al., 2020; Boothe, 2021; Boothe, 2019; Abelson et al., 2007), the Canadian Agency for Drugs and Technologies in Health (CADTH) HTA body and its oncology branch, the pan-Canadian Oncology Drug Review (pCODR), have institutionalised PPI at multiple levels. Patient advocacy groups, registered with CADTH, submit patient group evidence using a standardised electronic questionnaire template and participate in interviews to complement the literature on patients’ values and experiences. Two public or patient representatives with voting rights sit on each expert committees of CADTH and pCODR (CDEC and pERC, respectively) and present patient submissions. Patients are also invited to provide feedback on draft recommendations, whereby feedback explaining how patient input contributed to the recommendations is published. Provincial HTA agencies, such as INESS in Quebec (Poder et al., 2020) and OHTAC in Ontario (Young et al., 2017; Boothe, 2021; Abelson et al., 2007), similarly include patient and public members on HTA boards and committees, patient group surveys, and use coordinators for the planning, recruitment, and coaching of patients.

#### 3.4.2 Structured practitioners: selective processes within established systems

Some countries in Europe and beyond demonstrate a moderate to high degree of institutionalisation, with structured but more selective forms of PPI within established systems. These processes are supported by dedicated resources and staff.

In Germany (Weeks et al., 2017; Menon and Stafinski, 2011; Kreis and Schmidt, 2013; Young et al., 2017; Gagnon et al., 2021), patient representatives participate in the Gemeinsamer Bundesausschuss (G-BA) board meetings and appraisal committees, where legally binding reimbursement decisions are decided, to propose topics for appraisal and contribute to discussions. However, they are not formal members and cannot vote. Patient organisations can also submit relevant evidence after the selected technology for appraisal is published on the website. The HTA agency IQWiG can be commissioned to provide evidence reports that generally invites patient representatives to provide input and comments on draft protocols and reviews, which are posted on their website for public consultation.

The Netherlands’ (Weeks et al., 2017; Menon and Stafinski, 2011; Gagnon et al., 2011; Scott et al., 2017; Lips et al., 2022; Gunn et al., 2023; Young et al., 2017) Zorginstituut Nederland (ZIN), which informs and makes formal reimbursement decisions, consults patient associations during the scoping phase to formulate relevant research questions and important outcomes, and invites them to comment on draft assessments. During the appraisal stage, ZIN reaches out to umbrella patient organisations to provide submissions to the agency, deliver a statement at public meetings, and include a patient representative on the appraisal committee.

In France (Weeks et al., 2017; Menon and Stafinski, 2011; Kreis and Schmidt, 2013; Na et al., 2023; Nabarette, 2018), the Haute Autorité de Santé (HAS) responsible for reimbursement decisions uses adapted HTAi PCIG templates for written submissions by patient associations, invites them at hearings, and includes representatives on its board and standing committee. A dedicated advisory group develops PPI processes, and when HAS’s opinion is published, feedback meetings with associations take place to discuss the strengths and weaknesses of the contribution, its influence on deliberations, and suggestions for the future.

In Belgium (Cleemput et al., 2015; Na et al., 2023; Cleemput et al., 2020), the Belgian Healthcare Knowledge Centre (KCE) advisory HTA body involves umbrella patient organisations on its board and consults patient groups at various HTA stages, ranging from targeted input on specific assessments to co-production in formulating recommendations. Patients are also involved in the call for proposals and KCE-sponsored clinical trials.

Australia (Menon and Stafinski, 2011; Lopes et al., 2016; Scott et al., 2017; Young et al., 2017; Messina and Grainger, 2012; Gagnon et al., 2021; Lopez Gousset et al., 2024) has integrated PPI mainly through the Pharmaceutical Benefits Advisory Committee (PBAC) and the Medical Services Advisory Committee (MSAC), both of which directly inform public reimbursement decisions. Both include a consumer representative on the advisory committees and accept online consultations from anyone to provide more information for consideration. When required, the committee request ‘consumer impact statements’ on patients’ quality of life and experience living with the condition.

Taiwan (Weeks et al., 2017; Scott et al., 2017; Chen et al., 2021; Single et al., 2021) provides an example of an Asian system increasingly aligned with international PPI standards. Patient groups are able to submit their experiences through an online platform that includes a guideline to assist the submission. During the assessment, the HTA division of the Center for Drug Evaluation (CDE) summarizes and incorporates these opinions into the HTA report. During the appraisal, patient representatives can participate without voting in the Pharmaceutical Benefits and Reimbursement Scheme (PBRS) committee meetings, which are responsible for reimbursement decisions, and disease-specific patient organizations may be invited to present their opinion, with a pre-meeting to discuss online provided input. They are also consulted on draft recommendation reports.

#### 3.4.3 Emerging practitioners: developing, expanding, and evolving systems

Several countries show moderate institutionalisation, with agencies introducing policies and processes to expand PPI more recently. Involvement often consists of evolving frameworks, moving from consultation towards early-stage participation, but implementation remains exploratory and lacks standardisation.

Spain’s (Toledo-Chávarri et al., 2020a; Toledo-Chávarri et al., 2020b; Toledo-Chávarri et al., 2019) network of regional HTA agencies (RedETS) illustrates a phased and gradual approach from pilot projects, capacity building, and annual evaluation to formalize PPI procedures and flowcharts for involving patient groups throughout protocol development, outcomes identification, assessment, and dissemination of patient-friendly versions. When technologies are expected to meaningfully affect patient experiences, values, or preferences, patient-based evidence is included through systematic literature reviews or primary studies. Patients are involved in assessing the need to collect or produce patient-based evidence, comment on the contextual relevance of existing literature, and provide direct input. Patients contribute primarily via consultation and communication, but do not participate in formal appraisals.

Brief discussions in literature highlight evolving PPI practices in several other European countries. Ireland (Gunn et al., 2023; Gagnon et al., 2021) includes patient representatives on advisory committees and informal consultations, while Austria (Gagnon et al., 2021) consults patients via qualitative research and participation on HTA boards. In the Americas, Colombia (Weeks et al., 2017) allows patient and the public to participate in committees and consult on the scope and outcomes of assessments. In the United States (Menon and Stafinski, 2011; Young et al., 2017), anyone can submit information, present views during review committees, and comment on draft recommendations, though consumer and patient representatives are mainly included in Patient-Centered Outcomes Research Institute (PCORI) research funding (Kreis and Schmidt, 2013).

#### 3.4.4 Early-stage or *ad hoc* initiators: minimal or inconsistent systems

This cluster included countries with low institutionalisation of PPI, with basic consultation-only practices or fragmented, *ad hoc*, or limited participation processes. Systemic structures and dedicated resources are lacking, and substantive efforts and investments are needed to develop these into established PPI practices.

Basic consultation-only processes occur in different countries. In Italy (Weeks et al., 2017; Gagnon et al., 2021; Jommi and Bertolani, 2018), patient associations contribute to regional working groups and patient data collection. Finland’s (Häme et al., 2016; Kleme et al., 2014; Tran et al., 2024; Gagnon et al., 2021) HTA agency Fimea and advisory body Council for Choices in Healthcare (COHERE) collect patient experiences via literature reviews, surveys and qualitative interviews, and invites patient organisations to comment on publicly available draft assessments. Poland (Weeks et al., 2017) also focusses on consultation mechanisms, with patients and the public able to review reports, and Denmark consulting patients via interviews (Gagnon et al., 2011). In Latin America, Brazil’s (Lopes ACF. et al., 2020; Castro and Elias, 2018; De Freitas Lopes et al., 2023; Holtorf et al., 2021) Conitec allows online public consultation on draft recommendations and occasionally holds public hearings, while Argentina’s (Alcaraz et al., 2025) HTA program organizes multi-stage public consultations to gather comments on HTAs. New Zealand (Menon and Stafinski, 2011; Young et al., 2017; Single et al., 2021) accepts patient representatives to submit information and comment on evaluation reports upon request.

Some Scandinavian and central European countries have established limited participation processes such as Sweden (Weeks et al., 2017; Young et al., 2017), Norway (Gunn et al., 2023), Switzerland (Young et al., 2017), and Romania (Weeks et al., 2017). They have introduced patients or the public as members of advisory or working group committees, but these practices are poorly documented and lack procedural clarity in the included body of literature.

### 3.5 Barriers to meaningful PPI

Despite considerable progress, the literature underscored numerous barriers limiting the depth and effectiveness of PPI in P&R procedures (Table 2). Insights from stakeholder interviews and focus group discussions complemented and contextualised these findings, providing in-depth perspectives on real-world experiences and practical barriers (overview of barriers in Table 4).

**TABLE 2:** Barriers mentioned in the scoping literature with their corresponding references.

Barrier	Total articles mentioning barrier	References
Resource constraints	N = 38	Whitty (2013), Menon and Stafinski (2011), Gauvin et al. (2010), Gagnon et al. (2011), Lopes et al. (2020a), Castro and Elias (2018), Abelson et al. (2016), Wortley et al. (2016), Cleemput et al. (2015), Häme et al. (2016), Gagnon et al. (2015), Atfeh et al. (2024), Stewart et al. (2021), Toledo-Chávarri et al. (2020b), Scott et al. (2017), Lips et al. (2022), Gagnon et al. (2014a), Hailey et al. (2013), Hashem et al. (2018), Lopes et al. (2020b), Messina and Grainger (2012), Johnson et al. (2024), Na et al. (2023), Cook et al. (2021), Gagnon et al. (2021), Bidonde et al. (2021), Mercer et al. (2020), Boothe, 2019; Nabarette (2018), Cleemput et al. (2020), Chen et al. (2021), Single et al. (2021), Toledo-Chávarri et al. (2019), Alcaraz et al. (2025), Jakab et al. (2023), Gauvin et al. (2011), Dimitrova et al. (2022), Desmet et al. (2024)
Unclear value and impact of PPI	N = 27	Dipankui et al. (2015), Gauvin et al. (2010), Castro and Elias (2018), Abelson et al. (2016), Lopes et al. (2016), Facey et al. (2010), Cleemput et al. (2015), Gagnon et al. (2015), Stewart et al. (2021), Scott et al. (2017), Lips et al. (2022), Hashem et al. (2018), Tran et al. (2024), Young et al. (2017), Messina and Grainger (2012), Johnson et al. (2024), Na et al. (2023), Bidonde et al. (2021), Boothe, 2021; Boothe (2019), Cleemput et al. (2020), Jakab et al. (2023), Gauvin et al. (2011), Dimitrova et al. (2022), Desmet et al. (2024), Pomey et al. (2020), Abelson et al. (2013)
Conflict of interest concerns	N = 25	Whitty (2013), Menon and Stafinski (2011), Gauvin et al. (2010), Cleemput et al. (2015), Gagnon et al. (2015), Gagnon et al. (2014a), Hashem et al. (2018), Na et al. (2023), Cook et al. (2021), Gagnon et al. (2021), Bidonde et al. (2021), Rocchi et al. (2015), Mercer et al. (2020), Boothe, 2021; Boothe (2019), Nabarette (2018), Cleemput et al. (2020), Jommi and Bertolani (2018), De Freitas Lopes et al. (2023), Jakab et al. (2023), Gauvin et al. (2011), Dimitrova et al. (2022), Desmet et al. (2024), Pomey et al. (2020), Janssens et al. (2018)
Recruitment challenges	N = 23	Whitty (2013), Gagnon et al. (2011), Castro and Elias (2018), Abelson et al. (2016), Lopes et al. (2016), Kleme et al. (2014), Gagnon et al. (2015), Stewart et al. (2021), Toledo-Chávarri et al. (2020b), Scott et al. (2017), Elvsaas et al. (2021), Lips et al. (2022), Gagnon et al. (2014a), Hailey et al. (2013), Lopes et al. (2020b), Johnson et al. (2024), Gagnon et al. (2021), Bidonde et al. (2021), Toledo-Chávarri et al. (2019), Jommi and Bertolani (2018), Jakab et al. (2023), Dimitrova et al. (2022), Janssens et al. (2018)
Representativeness and diversity concerns	N = 22	Whitty (2013), Menon and Stafinski (2011), Gauvin et al. (2010), Abelson et al. (2016), Lopes et al. (2016), Cleemput et al. (2015), Häme et al. (2016), Gagnon et al. (2015), Toledo-Chávarri et al. (2020b), Scott et al. (2017), Elvsaas et al. (2021), Gagnon et al. (2014a), Na et al. (2023), Bidonde et al. (2021), Mercer et al. (2020), Boothe (2021), Cleemput et al. (2020), Toledo-Chávarri et al. (2019), De Freitas Lopes et al. (2023), Jakab et al. (2023), Dimitrova et al. (2022), Janssens et al. (2018)
Complexity of HTA reports	N = 21	Dipankui et al. (2015), Gauvin et al. (2010), Gagnon et al. (2011), Castro and Elias (2018), Lopes et al. (2016), Cleemput et al. (2015), Gagnon et al. (2015), Atfeh et al. (2024), Toledo-Chávarri et al. (2020a), Gagnon et al. (2014a), Hashem et al. (2018), Lopes et al. (2020b), Johnson et al. (2024), Na et al. (2023), Cook et al. (2021), Bidonde et al. (2021), Nabarette (2018), Cleemput et al. (2020), Jommi and Bertolani (2018), Gauvin et al. (2011), Dimitrova et al. (2022)
Methodological gaps	N = 19	Weeks et al. (2017), Menon and Stafinski (2011), Gauvin et al. (2010), Lopes et al. (2020a), Castro and Elias (2018), Facey et al. (2010), Gagnon et al. (2015), Toledo-Chávarri et al. (2020a), Gagnon et al. (2014a), Bidonde et al. (2021), Poder et al. (2020), Boothe, 2021; Boothe (2019), Cleemput et al. (2020), De Freitas Lopes et al. (2023), Jakab et al. (2023), Gauvin et al. (2011), Dimitrova et al. (2022), Janssens et al. (2018)
Limited experience	N = 18	Whitty (2013), Dipankui et al. (2015), Gagnon et al. (2011), Abelson et al. (2016), Wortley et al. (2016), Toledo-Chávarri et al. (2020b), Scott et al. (2017), Gagnon et al. (2014a), Hashem et al. (2018), Lopes et al. (2020b), Johnson et al. (2024), Bidonde et al. (2021), Chen et al. (2021), Single et al. (2021), Jakab et al. (2023), Dimitrova et al. (2022), Pomey et al. (2020), Athanasakis et al. (2021)
Political sensitivity	N = 7	Gauvin et al. (2010), Abelson et al. (2016), Na et al. (2023), Gagnon et al. (2021), De Freitas Lopes et al. (2023), Gauvin et al. (2011), Janssens et al. (2018)
Confidentiality	N = 7	Lopes et al. (2016), Scott et al. (2017), Hailey et al. (2013), Boothe, 2021; Nabarette (2018), Dimitrova et al. (2022), Pomey et al. (2020)
Workload	N = 5	Gagnon et al. (2015), Toledo-Chávarri et al. (2020b), Elvsaas et al. (2021), Gagnon et al. (2014a), Boothe (2019)

Resource constraints (n = 38) are the most frequently reported barrier. Both patient organisations and HTA agencies often lacked sufficient funding, time, staff capacity, and methodological expertise. The demand for rapid assessments with tight timelines or late notice for input conflicted with the time needed for high-quality engagement. Formal processes were also missing in many settings, leading to inconsistent and superficial input. Interviewees mainly stressed that patient organisations, which are often based on voluntary work and operate with limited organisational support, have little time to participate, noting that: *“Resources are a barrier since there is a push to approve and reimburse medicines faster, which makes it difficult to involve people in a meaningful way.” (Policymaker_PM2).*

A second major concern is the unclear value and impact of PPI (n = 27). Contributions were sometimes dismissed as anecdotal, subjective, biased, or insufficiently scientific, limiting their legitimacy relative to clinical and economic evidence. Due to limited evidence of how PPI was weighted and influenced decision-making, the added value was not always clear. This uncertainty fostered tokenism and questioned the willingness and openness to listen to patients’ views. This barrier was also most frequently mentioned during the interviews, where PPI was perceived as subjective, emotional, or lacking scientific weight. Some expressed concerns about whether voting rights for patient representatives were appropriate or feasible: *“Why would patients say no to reimbursement?” (Industry_IND3)*, while patients felt their perspectives were undervalued: “*They are not taking into consideration what impact it has on patients’ lives. We’re not invisible, but we have been made invisible by our system.” (Patient organisation_PO5)*. Several participants noted that financial considerations continued to dominate decisions, further limiting the space for patient perspectives and making PPI a tick-box exercise.

Conflict of interest concerns (n = 25) further complicate the landscape. Patient organisations partially or largely financed by industry raised fear about their objectivity, neutrality, and independence, since they may act as lobbyists and be biased towards industry positions, interests, and needs. These concerns about neutrality and credibility led some agencies to exclude patient groups funded more than 50% by a single manufacturer (Rocchi et al., 2015), further limiting the pool of potential contributors. Mostly industry participants underscored these issues, noting *“It is a boomerang effect for industry to financially support patient organisations as afterwards they get penalized for receiving funding and their voice no longer counts.” (IND4)*.

Recruitment challenges (n = 23) compound these problems. Agencies struggled to find, identify, and select suitable representatives who are knowledgeable, experienced, available, and able to convey perspectives effectively. Recruitment was further impeded by the lack of existing or willing disease-specific patient organisations and limited awareness of involvement opportunities. Interview participants highlighted these concerns, particularly for rare diseases and smaller or fragmented patient organisations.

Representativeness and diversity (n = 22) are other points of concerns, with uncertainties arising as to whether patients could represent the broader patient community. Narrow groups of engaged ‘professionalized’ patient representatives dominated smaller and less active patient organisations. Engagement tended to involve well-educated, younger, and English-speaking individuals, underrepresenting marginalized or socio-economically disadvantaged populations. Heterogeneity within patient populations, specific regional or local contexts, and cultural differences added further complexity to the generalisability and legitimacy to represent the diversity of patient perspectives. Interview participants further stressed that patient representatives should capture *“the greater common denominator of the opinions of the patient group” (IND1)* and act as spokespersons for all patients, including those *“who do not speak up” (PM5)*. They emphasised the need for disease-specific input, especially for rare conditions, rather than relying solely on non-disease specific umbrella patient organisations, and highlighted challenges in aggregating perspectives from multiple organisations or patients with multiple conditions and combinations of treatments. In this regard, patient experience data (patient-based data such as patient-reported outcomes, experience, patient input, and patient preferences) collected through qualitative and quantitative methods are deemed needed. Practical barriers, such as digital literacy and accessibility for older or disabled patients, were also noted for completing patient submissions.

The complexity of HTA reports (n = 21) poses another obstacle. Many participants reported difficulty understanding medical and technical jargon, which hindered their ability to contribute fully and equally. Lengthy documents and unfamiliarity with P&R and HTA procedures left some participants feeling uncomfortable or intimidated, a feeling sometimes amplified by numerical inferiority or power inequities. Ambiguity about roles and expectations of PPI left patients unsure of their purpose, while agencies feared that diverging perspectives could slow down procedures and make it even more complex. Interview participants emphasised the readability issues and that patients may feel overwhelmed, with one explaining: *“Patients are caught in the middle, realizing how powerless they are in committee meetings. Then I wonder, will not adding a patient representative just complicate and delay the process for what added value?” (PO2)*. Methodological gaps (n = 19), including the lack of guidance on rigorous approaches, appropriate methods, and standardised tools, further limit quality and consistency of PPI, especially related to qualitative research methods. Limited experience (n = 18) in conducting, collecting, interpreting, and using high-quality PPI adds further complexity.

Other barriers include the political sensitivity (n = 7) of P&R decisions and leadership changes impacting long-term participatory principles. Confidentiality (n = 7) agreements protecting proprietary information about the technology under assessment restrict transparency, interaction, and sharing of committee deliberations. Some studies raised concerns about participants feeling an overwhelming additional and demanding workload (n = 5) to collect high-quality submissions, prepare meetings, and review drafts, especially for participants involved throughout the whole process. This pressure often resulted from insufficiently planned PPI practices. Interviewees confirmed: *“It requires considerable work given that it is often not patients’ full-time job” (PO1)*.

Finally, the literature covers other barriers, including ethical issues (e.g., inclusion of vulnerable patients) (Pomey et al., 2020), lack of clarity in PPI definitions (Bidonde et al., 2021; Boothe, 2019), variable transparency in processes (Lopes et al., 2016; Lips et al., 2022; Lopes E. et al., 2020), lack of feedback on PPI practices (Lopes et al., 2016; Gagnon et al., 2014a; Johnson et al., 2024; Mercer et al., 2020), limited influence due to too late involvement (Dipankui et al., 2015), different regulations in different countries (Cook et al., 2021), and the absence of mandatory requirements in (local) HTA guidelines (Jakab et al., 2023). One participant also noted: *“Patients might unconsciously adapt their input to what they believe committee members want to hear” (Focus group_FG1_patient_P8)*.

### 3.6 Enablers for meaningful PPI

Alongside the identified barriers, both the scoping review (Table 3) and stakeholders’ perspectives revealed a variety of enablers to strengthen and sustain PPI in P&R procedures (overview of enablers in Table 4).

**TABLE 3:** Enablers mentioned in the scoping literature with their corresponding references.

Enabler	Total articles mentioning enabler	References
Education and training	N = 27	Menon and Stafinski (2011), Gauvin et al. (2010), Gagnon et al. (2011), Lopes et al. (2016), Facey et al. (2010), Cleemput et al. (2015), Dipankui et al. (2014), Häme et al. (2016), Gagnon et al. (2015), Toledo-Chávarri et al. (2020b), Scott et al. (2017), Gagnon et al. (2014a), Tran et al. (2024), Messina and Grainger (2012), Cook et al. (2021), Rocchi et al. (2015), Lopez Gousset et al. (2024), Single et al. (2021), Toledo-Chávarri et al. (2019), Jommi and Bertolani (2018), Jakab et al. (2023), Desmet et al. (2024), Pomey et al. (2020), Athanasakis et al. (2021), Rasburn et al. (2021), Georgieva and Yanakieva (2021), Gagnon et al. (2014b)
Resource investment	N = 22	Dipankui et al. (2015), Weeks et al. (2017), Menon and Stafinski (2011), Abelson et al. (2016), Facey et al. (2010), Atfeh et al. (2024), Toledo-Chávarri et al. (2020b), Moran and Davidson (2011), Scott et al. (2017), Lips et al. (2022), Kreis and Schmidt (2013), Johnson et al. (2024), Cook et al. (2021), Lopez Gousset et al. (2024), Alcaraz et al. (2025), Jakab et al. (2023), Gauvin et al. (2011), Desmet et al. (2024), Janssens et al. (2018), Rasburn et al. (2021), Georgieva and Yanakieva (2021), Gagnon et al. (2014b)
Clear communication and accessible guidance	N = 21	Menon and Stafinski (2011), Abelson et al. (2016), Facey et al. (2010), Häme et al. (2016), Toledo-Chávarri et al. (2020b), Scott et al. (2017), Gagnon et al. (2014a), Kreis and Schmidt (2013), Lopes et al. (2020b), Messina and Grainger (2012), Johnson et al. (2024), Cook et al. (2021), Gagnon et al. (2021), Poder et al. (2020), Lopez Gousset et al. (2024), Toledo-Chávarri et al. (2019), Jommi and Bertolani (2018), Dimitrova et al. (2022), Janssens et al. (2018), Rasburn et al. (2021), Georgieva and Yanakieva (2021)
Recruitment strategies	N = 21	Whitty (2013), Gagnon et al. (2011), Lopes et al. (2016), Cleemput et al. (2015), Dipankui et al. (2014), Häme et al. (2016), Gagnon et al. (2015), Atfeh et al. (2024), Toledo-Chávarri et al. (2020b), Elvsaas et al. (2021), Gagnon et al. (2014a), Kreis and Schmidt (2013), Messina and Grainger (2012), Johnson et al. (2024), Gagnon et al. (2021), Lopez Gousset et al. (2024), Toledo-Chávarri et al. (2019), Alcaraz et al. (2025), Jakab et al. (2023), Pomey et al. (2020), Janssens et al. (2018)
Feedback and evaluation mechanisms	N = 18	Gagnon et al. (2015), Atfeh et al. (2024), Toledo-Chávarri et al. (2020b), Moran and Davidson (2011), Scott et al. (2017), Gagnon et al. (2014a), Tran et al. (2024), Messina and Grainger (2012), Livingstone et al. (2021), Mercer et al. (2020), Boothe, 2019; Nabarette (2018), Lopez Gousset et al. (2024), Single et al. (2021), Gauvin et al. (2011), Abelson et al. (2013), Rasburn et al. (2021), Gagnon et al. (2014b)
Transparency	N = 15	Gauvin et al. (2010), Facey et al. (2010), Cleemput et al. (2015), Toledo-Chávarri et al. (2020b), Lopes et al. (2020b), Tran et al. (2024), Messina and Grainger (2012), Johnson et al. (2024), Rocchi et al. (2015), Boothe, 2019; Nabarette (2018), Cleemput et al. (2020), Lopez Gousset et al. (2024), Single et al. (2021), Gauvin et al. (2011)

**TABLE 4:** Overview of barriers and enablers for patient and public involvement in pricing and reimbursement decision-making.

Category	Barriers	Enablers
Structural and organisational	• Limited capacity, tools, and experience • Unclear value and influence of PPI • Confidentiality limiting transparency • High workload, tight timelines • Political sensitivity, ethical uncertainty	• Dedicated staff and formalised processes • Early involvement and adequate preparation time • Adequate logistical and financial support for participation • Institutionalisation of PPI
Representation and recruitment	• Over-representation of “professionalised” patients • Under-representation of vulnerable or marginalised groups • Disease heterogeneity • Fragmented or absent disease-specific patient organisations, e.g., in rare diseases • Digital, literacy, and accessibility barriers • Difficulty finding knowledgeable, diverse, and available representatives • Limited awareness, late notification of possibilities	• Mixed recruitment strategies: targeted and open call • Targeted outreach to underserved communities • Registries or network of trained patient contributors • Collaboration with umbrella organisations nationally and cross-border • Early notification of upcoming submissions
Procedural and technical	• Complexity of HTA language, methods and processes • Ambiguous roles and unclear expectations • Limited methodological support, e.g., for qualitative evidence	• Training for both patients and P&R agency members • Plain-language summary, glossary, guidance documents • Tailored PPI approaches (focus groups, interviews, multi-criteria decision-making, discrete choice experiments, value-based pricing models)
Financial and transparency	• Insufficient compensation for participation • Concerns about industry funding and perceived bias • Limited insights into how patient input is used • Lack of follow-up to contributors	• Public funding mechanisms and fair compensation • Transparent declaration of interests • Clear documentation of PPI contributions • Feedback loops improving motivation and trust

Education and training (n = 27) emerged as key enablers. Effective training programs should target both HTA agencies, to improve the understanding of PPI’s value, methods, and good practices, and patients and the public, to build basic knowledge of treatment effects, health economics, drug life cycle, and P&R procedures in order to facilitate participation. The public involvement programme at NICE and SMC in the United Kingdom provided preparatory workshops and pre-meeting briefings before committee meetings, as well as peer mentoring between experienced and new participants (Weeks et al., 2017; Rasburn et al., 2021). Such initiatives were considered to mitigate knowledge gaps, enhance confidence, and improve the quality of contributions. Interviewees strongly reinforced the need for *“patients and patient organisations to understand the entire medicines pathway.” (PM1)* with training helping them to become more neutral, knowledgeable, and accountable.

Moreover, resource investment (n = 22) to institutionalise PPI was also considered essential. Agencies with dedicated, qualified, and trained PPI units or coordinators, such as G-BA’s patient support team in Germany (Kreis and Schmidt, 2013), reported more consistent and high-quality participation. Supportive materials, such as submission templates, guidance documents, webinars, and clear meeting agendas, facilitated participation, as indicated by Canada’s experiences (Weeks et al., 2017). Participants reinforced the need for *“dedicated staff to institutionalise the involvement of patients and the public.” (PM4)*. Also, formalized processes are needed to define roles and operational procedures for ensuring sustainability and consistency. Adequate time allocation was also considered critical to support early involvement, preparation, realistic timelines, and flexible scheduling. Financial compensation and reimbursement for travel costs validated participation as professional work rather than voluntary goodwill. Practices varied across agencies, with proportional income-based compensation at G-BA, reimbursement for meeting attendance at HAS, and equal honoraria for patient experts and committee members at NICE (Kreis and Schmidt, 2013). Interviewees strongly supported fair compensation to avoid inequity: *“If people spend time to provide input during their job, they should at least be compensated for the loss of income associated with it. That’s just fair. Otherwise, no one will want to do it. If you pay peanuts, you get monkeys.” (IND1)*. They also emphasised that payments should reflect fair market value and be proportional to the effort without becoming the primary motivator. Some also noted that patient organisations already receiving governmental funding should not be additionally compensated as PPI is part of their core mandate.

Clear communication and accessible guidance (n = 21) further improved engagement. Providing information about P&R processes and the specific role of PPI clarified its purpose and format. Plain-language, patient-friendly versions of HTA reports, including glossaries and explanatory introductions, facilitated comprehension and resulted in higher participant satisfaction. Multichannel communication through online platforms, webinars, newsletters, and social media expanded reach and awareness of engagement opportunities. Interviewees stressed the importance of raising awareness on opportunities for input and the need for early communication on ongoing procedures to enable timely participation.

Recruitment strategies (n = 21) that combined variety of sources, such as open calls and targeted outreach to harder-to-reach communities enhanced diversity and representation. Strengthening partnerships with patient organisations, according to Finland’s practices (Kleme et al., 2014), and maintaining a database or registry of engaged patient contributors allowed agencies to mobilize participants quickly and effectively. Interviewees supported the practicality of maintaining formal networks, similar to the EMA. For rare diseases or fragmented patient groups, participants highlighted *“the need to collaborate with European-level or cross-border associations” (PO3)*.

Feedback and evaluation mechanisms (n = 18) played a crucial role in sustaining motivation and trust. Explaining how patient and public input was used and incorporated into decision-making and evaluating engagement processes positively impacted satisfaction and quality of input. Interviewees emphasised the need for continuous evaluation of the quality and impact of PPI processes, noting that initial procedures may require adjustments and improvements: *“You can have a really good idea beforehand but then it turns out to work very badly in practice” (IND1)*. They also underscored that more active and detailed feedback is needed on which data were considered, how it influenced the decision, and what the outcome was so patients can learn from this in future cases.

The need for more transparency (n = 15) was also repeatedly emphasised in literature as an enabler for increased legitimacy, accountability, and acceptability of decision-making. Insights into PPI processes and its outcomes through the publication of meeting minutes, clear mention of PPI contributions in reports, holding committee meetings in public (such as NICE), and disclosing conflict-of-interest declarations (such as Scotland) were considered good practices in the literature. Participants of the interviews stressed the importance of transparent declarations of interest in order to deal with them in the right way: *“There is a difference between declaring an interest and having a conflict. It is just about transparency.” (PM2)*. They highlighted that PPI itself can increase transparency over time as patients better understand the rationale, criteria, and reasoning behind decisions.

Other enablers mentioned in the literature include appropriate financial and logistical support for patient organisations (Abelson et al., 2016; Cleemput et al., 2015; Messina and Grainger, 2012; Mercer et al., 2020; Nabarette, 2018; Jakab et al., 2023), organisational culture supporting participation (Gagnon et al., 2011; Abelson et al., 2016; Cleemput et al., 2015; Dipankui et al., 2014; Bidonde et al., 2021), earlier involvement strategies (Häme et al., 2016; Young et al., 2017; Messina and Grainger, 2012), PPI assessment criteria established through consensus (Elvsaas et al., 2021; Cleemput et al., 2020; Single et al., 2021), and innovative approaches to incorporate patients’ values (e.g., multi-criteria decision-making, discrete choice experiments, value-based pricing models) (Gagnon et al., 2011; Wortley et al., 2016; Lopes et al., 2016; Berglas et al., 2016). Interviews added to give patient organisations lead time for data collection and tailor engagement methods to disease contexts: *“Additional dialogue beside patient submissions could capture more underlying perspectives in areas with greater relevance, for instance for rare diseases or areas with limited clinical evidence.” (PO3)*.

### 3.7 Evaluation and impact of PPI

Evidence of measurable PPI impact remains limited, but several tangible outcomes have been reported in the included body of literature. PPI influenced HTA protocols and its objectives, scope, population, and relevant outcome measures, as well as the planning and conduct of assessments and the interpretation of evidence to inform decision-making (Whitty, 2013; Dipankui et al., 2015; Kleme et al., 2014; Toledo-Chávarri et al., 2020a; Stewart et al., 2021; Elvsaas et al., 2021; Gunn et al., 2023; Tran et al., 2024; Young et al., 2017; Berglas et al., 2016; Boothe, 2019; Lopez Gousset et al., 2024; Abelson et al., 2013). PPI provided a unique perspective on patient experiences and dynamics, helping to balance clinical and economic evidence with patient-related considerations and contextualisation. It broadened the evidence base and refocuses the discussion on patient concerns during committee meetings, e.g., by presenting new data such as costs, demonstrating data limitations, stressing patient acceptability, or highlighting inequalities or special needs of subpopulations (Dipankui et al., 2015; Dipankui et al., 2014; Stewart et al., 2021; Scott et al., 2017; Elvsaas et al., 2021; Kreis and Schmidt, 2013; Livingstone et al., 2021; Lopez Gousset et al., 2024). Agencies with a longer history of PPI reported broader cultural effects, with staff becoming more aware of the patient perspective and feeling more comfortable and confident in decision-making due to improved credibility, legitimacy, and social accountability (Kreis and Schmidt, 2013; Livingstone et al., 2021; Boothe, 2019; Lopez Gousset et al., 2024; Pomey et al., 2020; Abelson et al., 2013).

Quantitative evidence on direct decision impact remains scarce. However, a notable example is a retrospective analysis of public consultations in Brazil, which demonstrated that Conitec’s preliminary recommendations were altered in 13% of cases following PPI, with a significantly higher likelihood of recommendation alteration when the number of contributions by patients and their families was high (defined as more than 100 contributions) (De Freitas Lopes et al., 2023). Overall, the impact of PPI was most frequently procedural and indirect, for example, by improving transparency, trust, and mutual understanding, rather than actually changing decisions (Tran et al., 2024). However, some studies indicated that some countries reported a low, limited, or unclear impact of PPI (Lips et al., 2022; Livingstone et al., 2021; Chen et al., 2021; Single et al., 2021). Due to the lack of systematic evaluation frameworks for PPI processes, impact, and participant satisfaction (such as Rowe’s model with criteria for assessing PPI effect in relation to effective construction and implementation), most evidence on PPI evaluation remains anecdotal and underreported (Gagnon et al., 2012; Gagnon et al., 2014b).

## 4 Discussion

### 4.1 International variation in PPI implementation

This mixed-methods study underscores that PPI in P&R procedures has gained global momentum, but reveals that implementation varies considerably in practice. Across healthcare systems, PPI implementation spans a spectrum, from basic information and limited or *ad hoc* consultation to more institutionalised participation.

Some countries have already established mature systems. England’s NICE, one of the earliest adopters, integrates PPI at multiple stages with deliberative mechanisms, such as citizen councils addressing social value judgements. Canada provides another example of systematic integration through structured patient submissions, interviews, and formal committee representation, supported by dedicated staff and explicit feedback loops. Other countries, mainly in Western Europe and Australia, have adopted structured yet more selective PPI practices. While PPI has been integrated into established P&R procedures, it often remains concentrated at specific stages rather than embedded comprehensively throughout the process. A growing number of emerging agencies has introduced PPI more recently and are steadily building experience, though mechanisms remain exploratory, evolving, uneven, and often not fully formalised. At the earliest stage, some countries, for example, in Eastern and Central Europe and Latin America, show only minimal, *ad hoc*, or inconsistent involvement with limited institutionalisation, standardisation, or supporting resources. The international network of agencies for HTA (INAHTA)’s surveys of 2005 and 2010 echoes these findings, revealing substantial heterogeneity in PPI approaches and emphasising the need for structured approaches (Hailey et al., 2013; Hailey and Nordwall, 2006). It is also important to note that not all HTA agencies directly make reimbursement or pricing decisions, with some primarily producing guidelines and recommendations. PPI tends to be most impactful, yet also most challenging, when embedded in agencies with authority to make actual P&R decisions.

Across countries, several recurring barriers were reported that shape this uneven and constrained real-world implementation of PPI in P&R procedures. Structural and organisational challenges included resource constraints, uncertainty about the purpose and influence of PPI, limited experience with PPI, concerns about confidentiality, and competing workloads. Representativeness remains a persistent concern due to the overreliance on “professionalised” patients and difficulties in reaching diverse or underrepresented groups. At the same time, recruitment is hindered by the lack of disease-specific organisations and limited awareness of PPI opportunities. Procedural and technical obstacles relate to the complexity of P&R terminology and processes, unclear roles and expectations, and insufficient methodological support for integrating experiential evidence. Transparency gaps and financial constraints, including concerns about conflict of interests further complicate PPI implementation in P&R. European Patients’ Forum (EPF) conducted a cross-European survey in 2013 among P&R agencies and patient organisations, underscoring these insights with lack of time, capacity, financial resources, and agreed methodologies, as well as the credibility of patient evidence as major challenges for PPI (EPF, 2013). A more recent study by HTAi, EUPATI, and EPF in 2024 confirms these challenges, highlighting a lack of awareness, capacity, and capability for patient involvement (Holtorf et al., 2024).

### 4.2 Alignment with global landscape and policy agendas

International organisations recognize the importance of meaningful and active PPI in P&R procedures. INAHTA has issued a position statement in 2021 affirming that PPI is an essential and valuable element to HTA, while the European Commission in its EU strategy for HTA envisages PPI throughout processes, including horizon scanning, scientific advice, and full assessments (INAHTA, 2021; EU Health Technology Assessment Network, 2014). Moreover, patient organisations such as EPF similarly call for greater transparency, adequate resourcing, and a shift in organisational culture to support meaningful PPI in EPF’s position statement on HTA and briefing on the EU regulatory landscape (EPF, 2018a; EPF, 2018b). Moreover, EPF’s recent recommendations for strengthening Joint Clinical Assessments (JCA) under the EU HTA Regulation emphasise the importance of structured pathways for PPI at the European level to increase the quality and relevance of JCAs, an issue of growing significance as JCA outputs will increasingly influence national P&R decisions (EPF, 2024).

Despite growing international interest, multiple stakeholders and organisations recognize the systemic deficiencies hampering PPI implementation. Industry-led analyses, including EFPIA’s multi-country review of 2024, similarly highlight fragmentation, selective and inconsistent practices, and insufficient feedback (EFPIA, 2024). While many agencies express commitment to PPI, few provide structured mechanisms or evaluate the effects of involvement. INAHTA’s survey data demonstrate wide heterogeneity in whether and how agencies operationalise involvement, with a minority having evaluation frameworks (Weeks et al., 2017; Hailey et al., 2013; Hailey and Nordwall, 2006). EPF’s cross-European survey underscores the same variation in type and level of PPI observed in this study, noting that while many agencies express commitment to PPI, few provide structured mechanisms or evaluate the effects of involvement. Moreover, the absence of systematic feedback loops identified in our study mirrors EPF’s findings that patients often do not know how their contributions influence decisions, a gap that contributes directly to perceptions of tokenism (EPF, 2013).

### 4.3 Towards more effective PPI integration

Besides barriers, this study identified several enablers to strengthen the effective and meaningful implementation of PPI in P&R procedures. Several international initiatives support these practices by providing tangible tools for improving PPI. For example, the HTAi PCIG offers methodological guidance and adaptable international plain-language summary templates, as introduced by Scotland’s SMC (Coombes et al., 2025). Moreover, a HTA glossary was developed to improve comprehensiveness of HTA terminology (INAHTA, 2025). These resources directly address one of the key barriers identified in our study, the complexity and inaccessibility of HTA materials, and reinforce our finding that clear communication tools are essential for equitable engagement. Moreover, training initiatives such as EUPATI and EUCAPA, alongside collaborative efforts such as HTAi’s 360° patient involvement programme, have significantly expanded training and knowledge opportunities for patients and P&R agencies (EUPATI, 2025; EUCAPA, 2025; Holtorf and Bertelsen, 2023).

Despite the growing availability of tools, guidance, and capacity-building initiatives, PPI remains inconsistently implemented, often hindered by practical barriers and by the lack of robust evidence on its tangible impact on decision-making. Based on our findings, a set of recommendations was developed to support more effective and meaningful PPI: (i) institutional commitment and leadership to establish PPI as a formal process with clearly defined expectations, purpose, roles, and scope of influence, (ii) dedicated resourcing, training, and support through adequate time, funding, and experience building, (iii) transparency and feedback to maintain two-way communication and build trust and legitimacy by demonstrating how PPI can shape decisions, (iv) methodological rigour and evaluation frameworks with standardised tools to capture, analyse, interpret, and evaluate experiential evidence consistently, (v) inclusivity and representativeness to ensure diverse perspectives and broad reach in participation opportunities via mixed recruitment strategies and tailored approaches, and (vi) capacity building to equip patients, public, and P&R agencies with the knowledge, skills, experience, and confidence to engage meaningfully and early on in decision-making. These practices collectively demonstrate that sustainable PPI in P&R is achievable when structural, organisational, procedural, technical, and financial enablers are aligned.

### 4.4 Strengths and limitations

This study offers a comprehensive and mixed-methods examination of PPI in P&R procedures, integrating evidence from a scoping literature review with stakeholder interviews and focus group discussions. This combination of methods allows a broad understanding of documented practices and perceived experiences. The international scope of the review across multiple countries and inclusion of diverse stakeholder groups in the qualitative study further enhance the exploration of concrete processes, good practices, concerns, and opportunities.

Nevertheless, some limitations apply. Inherent to scoping literature reviews, the aim was breadth rather than completeness. As such, the final sample of articles is not exhaustive nor representative of all publications on the topic. Through careful and systematic selection, it captures the diversity of PPI across countries. Yet, the available literature predominantly reflects experiences from high-income countries with established HTA and P&R systems, while evidence from low- and middle-income countries remains limited. In addition, relatively few quantitative studies assess the measurable impact of PPI on P&R procedures, suggesting a broader gap in the existing scientific evidence rather than the scope of the review itself. The variation in terminology, inconsistent reporting standards, and limited transparency in describing PPI processes across countries also constrained the ability to fully map or compare existing PPI practices. Additionally, some included studies may reflect older practices that could have changed or that are not accurately reported, as more recent developments may not yet be documented in scientific publications and could instead be reflected in statements on national institutional websites. However, this study focused on reported practices rather than directly observing PPI processes. As a result, discrepancies may exist between formal procedures, stakeholders’ perceptions, and actual decision-making.

Interview and focus group discussions provided additional depth into real-world insights on experiences and perceptions through a systematic approach. Participants were recruited through purposive sampling, reflecting both expertise and accessibility. The sampling aimed to capture a diversity of perspectives rather than achieve statistical representativeness, but may have contributed to overrepresentation of highly engaged, positive bias towards PPI, or well-informed stakeholders. Their perspectives may not fully capture the wide range of views on PPI practices. To mitigate this, initial contacts were selected across diverse stakeholder groups. The number of participants was appropriate for thematic analysis, but the findings are not intended to be generalisable. Instead, they provide insight into patterns and shared challenges and opportunities across stakeholder groups.

## 5 Conclusion

This study demonstrates that while the degree of PPI in P&R remains uneven and inconsistently implemented across countries, the direction of change is clear, with healthcare systems gradually shifting from limited information and symbolic consultation towards more structured participation and partnership. While countries differ in pace, level, and depth of implementation, there is a shared increasing consensus on the value, necessity, and relevance of patient perspectives in P&R decision-making. Our findings highlight that meaningful and sustainable PPI implementation requires the alignment of multiple structural, organisational, procedural, technical, and financial enablers. Across countries and stakeholder groups, several core recommendations consistently emerge as essential for effective implementation. These include clear institutional commitment and leadership, dedicated resources and support with clearly defined roles, expectations, and purpose, transparency and systematic feedback, methodological rigour and evaluation frameworks, inclusive and representative approaches, and capacity building for both patients and P&R agencies. When these enablers are absent or insufficiently coordinated, involvement risks remaining fragmented, symbolic or tokenistic, limiting its ability to meaningfully inform and influence decision-making. However, when in place and mutually reinforcing, PPI can become a substantive driver of more patient-centred, transparent, and socially accountable P&R procedures.

## Data Availability

The raw data supporting the conclusions of this article will be made available by the authors, without undue reservation.
