# Supplementary material for: International practices, barriers, and enablers for patient and public involvement in pricing and reimbursement decision-making: a mixed-methods study
Source: Front Pharmacol. 2026 Apr 21;17:1791567. doi: 10.3389/fphar.2026.1791567 (PMC13139171; doi:10.3389/fphar.2026.1791567)
Supplement: Supplementary file 1 [file Supplementaryfile1.docx]

Supplementary Materials

Supplementary materials 1: Search strategy for scoping literature review

**Scoping literature review**

**Stage 1: Identifying the research question**

Research question: How is patient and public involvement (PPI) described in the existing scientific literature regarding pricing and reimbursement decisions for health technologies across different countries worldwide to identify key PPI strategies?

**Stage 2: Identifying relevant studies**

**Databases**

- PubMed (via NCBI) – including MEDLINE
- Embase (via embase.com)
- Scopus (via scopus.com)

**Concepts**

- Patient and public involvement
- Pricing and reimbursement procedure

Search conducted on 17 December 2024

No limitations on time span and language

**Terms per concept**

| **Concept** | **PubMed** | **Embase** | | **Scopus** |
| --- | --- | --- | --- | --- |
| **C1** | "Patient Participation"[Mesh] OR "patient advocacy"[Mesh] OR "patient participation"[tiab:~3] OR "patient participations"[tiab:~3] OR "patients participation"[tiab:~3] OR "patients participations"[tiab:~3] OR "public participation"[tiab:~3] OR "public participations"[tiab:~3] OR "patient participating"[tiab:~3] OR "patients participating"[tiab:~3] OR "public participating"[tiab:~3] OR "patient participated"[tiab:~3] OR "patients participated"[tiab:~3] OR "public participated" [tiab:~3] OR "patient participate"[tiab:~3] OR "patients participate"[tiab:~3] OR "public participate"[tiab:~3] OR "patient participates"[tiab:~3] OR "patients participates"[tiab:~3] OR "public participates"[tiab:~3] OR "patient involvement"[tiab:~3] OR "patients involvement"[tiab:~3] OR "public involvement"[tiab:~3] OR "patient involving"[tiab:~3] OR "patients involving"[tiab:~3] OR "public involving"[tiab:~3] OR "patient involved"[tiab:~3] OR "patients involved"[tiab:~3] OR "public involved"[tiab:~3] OR "patient involve"[tiab:~3] OR "patients involve"[tiab:~3] OR "public involve"[tiab:~3] OR "patient involves"[tiab:~3] OR "patients involves"[tiab:~3] OR "public involves"[tiab:~3] OR "patient engagement"[tiab:~3] OR "patients engagement"[tiab:~3] OR "public engagement"[tiab:~3] OR "patient engage"[tiab:~3] OR "patients engage"[tiab:~3] OR "public engage"[tiab:~3] OR "patient engages"[tiab:~3] OR "patients engages"[tiab:~3] OR "public engages"[tiab:~3] OR "patient engaged"[tiab:~3] OR "patients engaged"[tiab:~3] OR "public engaged"[tiab:~3] OR "patient input"[tiab:~3] OR "patients input"[tiab:~3] OR "public input"[tiab:~3] OR "patient voice"[tiab:~3] OR "patients voice"[tiab:~3] OR "public voice"[tiab:~3] OR "patient voices"[tiab:~3] OR "patients voices"[tiab:~3] OR "public voices"[tiab:~3] OR "patient role"[tiab:~3] OR "patients role"[tiab:~3] OR "patient roles"[tiab:~3] OR "patients roles"[tiab:~3] OR "public role"[tiab:~3] OR "public roles"[tiab:~3] OR "patient perspective"[tiab:~3] OR "patients perspective"[tiab:~3] OR "patient perspectives"[tiab:~3] OR "patients perspectives"[tiab:~3] OR "public perspective"[tiab:~3] OR "public perspectives"[tiab:~3] OR "patient* organization*"[tiab] OR "patient* organisation*"[tiab] OR "patient* advisory"[tiab] OR "public advisory"[tiab] OR "patient* advocacy"[tiab] OR "public advocacy"[tiab] OR "patient* representative*"[tiab] OR "public representative*"[tiab] | 'patient participation'/exp OR 'patient advocacy'/exp OR ('patient*' NEAR/4 ('participat*' OR 'involv*' OR 'engag*')):ti,ab,kw OR ('public' NEAR/4 ('participat*' OR 'involv*' OR 'engag*')):ti,ab,kw OR ('patient*' NEAR/4 'input'):ti,ab,kw OR ('public' NEAR/4 'input'):ti,ab,kw OR ('patient*' NEAR/4 'role*'):ti,ab,kw OR ('public' NEAR/4 'role*'):ti,ab,kw OR ('patient*' NEAR/4 'voice*'):ti,ab,kw OR ('public' NEAR/4 'voice*'):ti,ab,kw OR ('patient*' NEAR/4 'perspective*'):ti,ab,kw OR ('public' NEAR/4 'perspective*'):ti,ab,kw OR ('patient* advocacy'):ti,ab,kw OR ('public advocacy'):ti,ab,kw OR ('patient* representative*'):ti,ab,kw OR ('public representative*'):ti,ab,kw OR ('patient* organization*'):ti,ab,kw OR ('patient* organisation*'):ti,ab,kw OR ('patient* advisory'):ti,ab,kw OR ('public advisory'):ti,ab,kw | | TITLE-ABS (("patient*" W/4 ("participat*" OR "involv*" OR "engag*" OR "input" OR "role*" OR "voice*" OR "perspective*")) OR ("public" W/4 ("participat*" OR "involv*" OR "engag*" OR "input" OR "role*" OR "voice*" OR "perspective*")) OR ("patient* advocacy") OR ("public advocacy") OR ("patient* representative*") OR ("public representative*") OR ("patient* organization*") OR ("patient* organisation*") OR ("patient* advisory") OR ("public advisory*")) OR AUTHKEY (("patient*" W/4 ("participat*" OR "involv*" OR "engag*" OR "input" OR "role*" OR "voice*" OR "perspective*")) OR ("public" W/4 ("participat*" OR "involv*" OR "engag*" OR "input" OR "role*" OR "voice*" OR "perspective*")) OR ("patient* advocacy") OR ("public advocacy") OR ("patient* representative*") OR ("public representative*") OR ("patient* organization*") OR ("patient* organisation*") OR ("patient* advisory") OR ("public advisory*")) |
| C2 | "Technology Assessment, Biomedical"[Mesh] OR "technology assessment*"[tiab] OR "reimbursement mechanisms"[Mesh] OR "reimbursement*"[tiab] OR "reimbursing*"[tiab] OR "pricing*"[tiab] OR "price*"[tiab] OR "health technology evaluation*"[tiab] OR "health technology appraisal*"[tiab] OR "HTA"[tiab] OR "HTAs"[tiab] | 'biomedical technology assessment'/exp OR 'reimbursement'/exp OR 'reimbursement*':ti,ab,kw OR 'reimbursing*':ti,ab,kw OR 'price*':ti,ab,kw OR 'pricing*':ti,ab,kw OR 'health technology evaluation*':ti,ab,kw OR 'health technology appraisal*':ti,ab,kw OR 'hta':ti,ab,kw OR 'htas':ti,ab,kw OR 'technology assessment*':ti,ab,kw | TITLE-ABS (("technology assessment*") OR ("reimbursement*") OR ("reimbursing*") OR ("price*") OR ("pricing*") OR ("health technology evaluation*") OR ("health technology appraisal*") OR ("hta") OR ("htas")) OR AUTHKEY (("technology assessment*") OR ("reimbursement*") OR ("reimbursing*") OR ("price*") OR ("pricing*") OR ("health technology evaluation*") OR ("health technology appraisal*") OR ("hta") OR ("htas")) | |

**Search string**

| **Database** | **Search string** | **Results** |
| --- | --- | --- |
| **C1 AND C2** | | |
| **PubMed** | ("Patient Participation"[Mesh] OR "patient advocacy"[Mesh] OR "patient participation"[tiab:~3] OR "patient participations"[tiab:~3] OR "patients participation"[tiab:~3] OR "patients participations"[tiab:~3] OR "public participation"[tiab:~3] OR "public participations"[tiab:~3] OR "patient participating"[tiab:~3] OR "patients participating"[tiab:~3] OR "public participating"[tiab:~3] OR "patient participated"[tiab:~3] OR "patients participated"[tiab:~3] OR "public participated" [tiab:~3] OR "patient participate"[tiab:~3] OR "patients participate"[tiab:~3] OR "public participate"[tiab:~3] OR "patient participates"[tiab:~3] OR "patients participates"[tiab:~3] OR "public participates"[tiab:~3] OR "patient involvement"[tiab:~3] OR "patients involvement"[tiab:~3] OR "public involvement"[tiab:~3] OR "patient involving"[tiab:~3] OR "patients involving"[tiab:~3] OR "public involving"[tiab:~3] OR "patient involved"[tiab:~3] OR "patients involved"[tiab:~3] OR "public involved"[tiab:~3] OR "patient involve"[tiab:~3] OR "patients involve"[tiab:~3] OR "public involve"[tiab:~3] OR "patient involves"[tiab:~3] OR "patients involves"[tiab:~3] OR "public involves"[tiab:~3] OR "patient engagement"[tiab:~3] OR "patients engagement"[tiab:~3] OR "public engagement"[tiab:~3] OR "patient engage"[tiab:~3] OR "patients engage"[tiab:~3] OR "public engage"[tiab:~3] OR "patient engages"[tiab:~3] OR "patients engages"[tiab:~3] OR "public engages"[tiab:~3] OR "patient engaged"[tiab:~3] OR "patients engaged"[tiab:~3] OR "public engaged"[tiab:~3] OR "patient input"[tiab:~3] OR "patients input"[tiab:~3] OR "public input"[tiab:~3] OR "patient voice"[tiab:~3] OR "patients voice"[tiab:~3] OR "public voice"[tiab:~3] OR "patient voices"[tiab:~3] OR "patients voices"[tiab:~3] OR "public voices"[tiab:~3] OR "patient role"[tiab:~3] OR "patients role"[tiab:~3] OR "patient roles"[tiab:~3] OR "patients roles"[tiab:~3] OR "public role"[tiab:~3] OR "public roles"[tiab:~3] OR "patient perspective"[tiab:~3] OR "patients perspective"[tiab:~3] OR "patient perspectives"[tiab:~3] OR "patients perspectives"[tiab:~3] OR "public perspective"[tiab:~3] OR "public perspectives"[tiab:~3] OR "patient* organization*"[tiab] OR "patient* organisation*"[tiab] OR "patient* advisory"[tiab] OR "public advisory"[tiab] OR "patient* advocacy"[tiab] OR "public advocacy"[tiab] OR "patient* representative*"[tiab] OR "public representative*"[tiab]) AND ("Technology Assessment, Biomedical"[Mesh] OR "technology assessment*"[tiab] OR "reimbursement mechanisms"[Mesh] OR "reimbursement*"[tiab] OR "reimbursing*"[tiab] OR "pricing*"[tiab] OR "price*"[tiab] OR "health technology evaluation*"[tiab] OR "health technology appraisal*"[tiab] OR "HTA"[tiab] OR "HTAs"[tiab]) |  |
| **Embase** | ('patient participation'/exp OR 'patient advocacy'/exp OR ('patient*' NEAR/4 ('participat*' OR 'involv*' OR 'engag*')):ti,ab,kw OR ('public' NEAR/4 ('participat*' OR 'involv*' OR 'engag*')):ti,ab,kw OR ('patient*' NEAR/4 'input'):ti,ab,kw OR ('public' NEAR/4 'input'):ti,ab,kw OR ('patient*' NEAR/4 'role*'):ti,ab,kw OR ('public' NEAR/4 'role*'):ti,ab,kw OR ('patient*' NEAR/4 'voice*'):ti,ab,kw OR ('public' NEAR/4 'voice*'):ti,ab,kw OR ('patient*' NEAR/4 'perspective*'):ti,ab,kw OR ('public' NEAR/4 'perspective*'):ti,ab,kw OR ('patient* advocacy'):ti,ab,kw OR ('public advocacy'):ti,ab,kw OR ('patient* representative*'):ti,ab,kw OR ('public representative*'):ti,ab,kw OR ('patient* organization*'):ti,ab,kw OR ('patient* organisation*'):ti,ab,kw OR ('patient* advisory'):ti,ab,kw OR ('public advisory'):ti,ab,kw) AND ('biomedical technology assessment'/exp OR 'reimbursement'/exp OR 'reimbursement*':ti,ab,kw OR 'reimbursing*':ti,ab,kw OR 'price*':ti,ab,kw OR 'pricing*':ti,ab,kw OR 'health technology evaluation*':ti,ab,kw OR 'health technology appraisal*':ti,ab,kw OR 'hta':ti,ab,kw OR 'htas':ti,ab,kw OR 'technology assessment*':ti,ab,kw) NOT ('conference abstract':it) |  |
| **Scopus** | TITLE-ABS (("patient*" W/4 ("participat*" OR "involv*" OR "engag*" OR "input" OR "role*" OR "voice*" OR "perspective*")) OR ("public" W/4 ("participat*" OR "involv*" OR "engag*" OR "input" OR "role*" OR "voice*" OR "perspective*")) OR ("patient* advocacy") OR ("public advocacy") OR ("patient* representative*") OR ("public representative*") OR ("patient* organization*") OR ("patient* organisation*") OR ("patient* advisory") OR ("public advisory*")) OR AUTHKEY (("patient*" W/4 ("participat*" OR "involv*" OR "engag*" OR "input" OR "role*" OR "voice*" OR "perspective*")) OR ("public" W/4 ("participat*" OR "involv*" OR "engag*" OR "input" OR "role*" OR "voice*" OR "perspective*")) OR ("patient* advocacy") OR ("public advocacy") OR ("patient* representative*") OR ("public representative*") OR ("patient* organization*") OR ("patient* organisation*") OR ("patient* advisory") OR ("public advisory*")) AND TITLE-ABS (("technology assessment*") OR ("reimbursement*") OR ("reimbursing*") OR ("price*") OR ("pricing*") OR ("health technology evaluation*") OR ("health technology appraisal*") OR ("hta") OR ("htas")) OR AUTHKEY (("technology assessment*") OR ("reimbursement*") OR ("reimbursing*") OR ("price*") OR ("pricing*") OR ("health technology evaluation*") OR ("health technology appraisal*") OR ("hta") OR ("htas")) |  |

The search strategy was developed in collaboration with a trained medical librarian from the 2Bergen Désiré Collen library of KU Leuven. Endnote was used as data management tool for managing records, keeping track of articles, and deduplicate articles from different databases.

**Stage 3: Study selection**

**Inclusion and exclusion criteria**

|  | **Search string** | **Results** |
| --- | --- | --- |
| Population | - Patients (including patient representative, members of patient organisations) and public members actively involved in the pricing and reimbursement procedure | - Studies not including patients (including patient representative, members of patient organisations) and public members - Patients (including patient representative, members of patient organisations) and public members that are not actively involved (for example; as participants in clinical studies or measuring patient reported outcomes, patient preferences, unmet needs,...) |
| Setting/context | - Pricing and reimbursement procedures, including health technology assessments in healthcare across various countries or non-country specific | - Articles not based on pricing and reimbursement procedures, including health technology assessments - Articles on technology assessment but not health related (environmental, industrial, digital, etc..) |
| Outcomes | - Studies based on the practical/organizational process of implementing patient and public in pricing and reimbursement procedures - Studies that identify barriers, opportunities, and best practices for patient and public involvement in pricing and reimbursement procedures - Studies that report recommendations for patient and public involvement in pricing and reimbursement procedures | - Other outcomes not related to the subject of describing the relation of patient involvement in pricing and reimbursement procedures |
| Publication type | - Peer reviewed journal articles | - Conference abstracts - Editorials - Comments - Opinion pieces / perspectives |

Two reviewers applied the inclusion and exclusion criteria to all included articles using Rayyan, with inclusion based on title abstract screening and final inclusion based on full text screening. Disagreements were resolved through discussion with a third reviewer.

**Stage 4: Charting the data**

The analysis was guided by the initial data extraction form based on existing literature on PPI in P&R procedures, while remaining open to additional concepts emerging from the included studies.

Data were charted in a Microsoft Excel data extraction form containing following descriptives:

- General information about the study
  - Title
  - Author(s)
  - DOI
  - Publication type
  - Year of publication
  - Study objectives
  - Study methodology
  - Results and conclusion of study
  - Disease-specific study: yes or no
- Country of interest
  - Country-specific study: yes or no
  - Described countries
  - Described pricing and reimbursement agencies
- Stakeholders involved
  - Amount of stakeholders
  - Characteristics
  - Type of input provided by stakeholder
- Selection and recruitment
  - Processes to select and recruit stakeholders
- Resources used for stakeholder involvement
  - Human resources
  - Materials
  - Structural resources
  - Time
  - Information
  - Economic resources
- Level of involvement
  - Description of level of involvement
  - Level of involvement: communication, consultation, participation
- Involvement framework used
  - Description of theoretical framework
- Process of stakeholder involvement
  - Strategy of involvement practices
  - Different steps applied
  - Type of data collected
  - Format of data collected
- Timing of stakeholder involvement
  - Pricing and reimbursement phase where involvement takes place
- Evolution of stakeholder involvement
  - Legal aspects
  - Years of experience
  - Future plans
- Barriers related to stakeholder involvement
  - Political
  - Workload
  - Complexity of pricing and reimbursement procedure
  - Socio-economic
  - Recruitment
  - Lack of evidence on the value of stakeholder involvement
  - Lack of resources
  - Lack of experience with stakeholder involvement
  - Ethical issues
  - Confidentiality issues
  - Conflict of interest issues
  - Methodological gaps
  - Representativeness issues
  - Other barriers
- Opportunities related to stakeholder involvement
  - Information and guidance
  - Education and training
  - Resource investment
  - Transparency
  - Feedback
  - Recruitment strategy
  - Support for patient organisations
  - Advocacy
  - Other
- Best practices
- Recommendations
- Reported impact of stakeholder involvement

Iterative process to test and refine the extraction form over time

**Stage 5: Collating, summarizing and reporting the results**

A thematic construction allowed to present a narrative synthesis of existing literature, including:

- Basic numerical, descriptive analysis of the extent, nature and distribution of studies included in the review, to shed light on the dominant areas of research
- Thematically organisation and analysis of literature

It allowed reviewers to provide an overview of all material reviewed

**Qualitative research**

Semi-structured interviews and focus group discussions with multi-stakeholders were organized to validate findings, provide valuable insights about the PPI, and improve the practical relevance of results. The preliminary findings from the reviews were used as a foundation to inform the consultation.

Supplementary materials 2: Study categories included in the scoping literature review


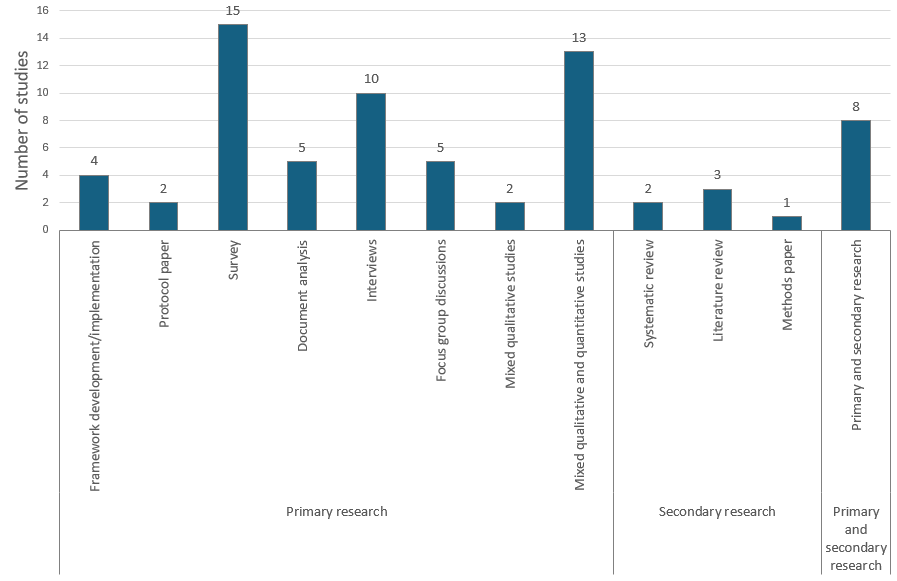


*Supplementary materials 3: Overview of article characteristics included in scoping literature review*

**Summary of article characteristics**

| **Article characteristics** | **Number of included articles (N=70)** |
| --- | --- |
| **Year of publication** | |
| 2006 | 1 |
| 2007 | 1 |
| 2010 | 2 |
| 2011 | 4 |
| 2012 | 3 |
| 2013 | 4 |
| 2014 | 3 |
| 2015 | 5 |
| 2016 | 4 |
| 2017 | 4 |
| 2018 | 4 |
| 2019 | 3 |
| 2020 | 13 |
| 2021 | 6 |
| 2022 | 3 |
| 2023 | 4 |
| 2024 | 5 |
| 2025 | 1 |
| **Disease scope of article** | |
| Disease specific (classification according to ICD-11) | 3 |
| ICD02 Neoplasms | 1 |
| ICD11 Diseases of the circulatory system | 1 |
| ICD24 Factors influencing health status or contact with health services | 1 |
| Non-disease specific | 67 |
| **Geographical countries represented in articles*** | |
| Country specific | 61 |
| Argentina | 2 |
| Australia | 10 |
| Austria | 4 |
| Belgium | 4 |
| Brazil | 6 |
| Bulgaria | 2 |
| Canada | 30 |
| Columbia | 1 |
| Croatia | 1 |
| Denmark | 4 |
| Finland | 6 |
| France | 9 |
| Germany | 11 |
| Greece | 2 |
| Hungary | 2 |
| Indonesia | 1 |
| Ireland | 3 |
| Israel | 3 |
| Italy | 5 |
| Japan | 1 |
| Kazakhstan | 1 |
| Lithuania | 3 |
| Luxembourg | 1 |
| Malaysia | 1 |
| Mexico | 2 |
| The Netherlands | 11 |
| New Zealand | 5 |
| Nigeria | 1 |
| North Macedonia | 1 |
| Norway | 4 |
| Poland | 4 |
| Romania | 2 |
| Serbia | 1 |
| Slovakia | 1 |
| Slovenia | 1 |
| South Africa | 1 |
| South Korea | 3 |
| Spain | 8 |
| Sweden | 4 |
| Switzerland | 1 |
| Taiwan | 5 |
| Turkey | 1 |
| Thailand | 1 |
| UK | 22 |
| Ukraine | 1 |
| USA | 5 |
| Non-country specific | 8 |

**More than one country may be included in the articles, resulting in a higher total amount of countries represented in the articles compared to the number of included articles. Countries mentioned in cross-national studies (for instance international surveys) were also included, although these studies did not specify responses at the specific individual-country level. Consequently, the list reflects geographic scope of countries mentioned in the literature, not the presence or absence of PPI practices.*

**Overview of identified articles**

| **Author** | **Year** | **Title** | **Geographical scope** | **Study design** |
| --- | --- | --- | --- | --- |
| **Gagnon et al.** | 2021 | Patient and public involvement in health technology assessment: update of a systematic review of international experiences | Canada, Australia, Italy, UK, Germany, Finland, Austria, Ireland, South Korea, Spain, The Netherlands, Norway, Lithuania, Poland | Systematic review |
| **Toledo-Chávarri et al.** | 2019 | Toward a Strategy to Involve Patients in Health Technology Assessment in Spain | Spain | Literature review, qualitative interviews and Delphi consultation |
| **Kleme et al.** | 2014 | Patient perspective in health technology assessment of pharmaceuticals in Finland | Finland | Literature review, qualitative interviews and focus group discussions |
| **Abelson et al.** | 2007 | Bringing ‘the public’ into health technology assessment and coverage policy decisions: From principles to practice | Canada | Framework development |
| **Abelson et al.** | 2016 | Public and patient involvement in health technology assessment: a framework for action | Canada | Website review, literature review, stakeholder dialogue |
| **Atfeh et al.** | 2024 | Involving Patients in Hospital‐Based Health Technology Assessment of Innovative Medical Devices: Adapting to a Specific Local Context and Lessons Learned From the Assessment of an Ex Vivo Perfusion System of Human Donor Hearts | France | Framework implementation |
| **De Freitas Lopes et al.** | 2023 | Does patient and public involvement impact public health decision making? A 10year retrospective analysis of public consultation in Brazil | Brazil | Document analysis |
| **Toledo-Chávarri et al.** | 2020 | Development of a decisional flowchart for meaningful patient involvement in Health Technology Assessment | Spain | Framework development |
| **Bidonde et al.** | 2021 | An institutional ethnographic analysis of public and patient engagement activities at a national health technology assessment agency | Canada | Observational research, document analysis, qualitative interviews |
| **Berglas et al.** | 2016 | Patients' perspectives can be integrated in health technology assessments: an exploratory analysis of CADTH Common Drug Review | Canada | Document analysis |
| **Young et al.** | 2017 | Exploring patient and family involvement in the lifecycle of an orphan drug: A scoping review | Australia, Canada, Denmark, Germany, The Netherlands, New Zealand, Sweden, Switzerland, UK, USA | Scoping literature review, stakeholder consultation |
| **Wortley et al.** | 2016 | What factors determine the choice of public engagement undertaken by health technology assessment decision making organizations? | Non-country specific | Framework development |
| **Whitty et al.** | 2013 | An international survey of the public engagement practices of health technology assessment organizations | Europe, Australasia, American continent | Quantitative survey |
| **Weeks et al.** | 2017 | Evaluation of patient and public involvement initiatives in health technology assessment: a survey of international agencies | UK, The Netherlands, Taiwan, Canada, Columbia, France, Germany, Italy, Luxembourg, Poland, Romania, Sweden | Quantitative survey |
| **Tran Minh et al.** | 2024 | Experiences of patient organizations' involvement in medicine appraisal and reimbursement processes in Finland: a qualitative study | Finland | Qualitative interviews |
| **Toledo-Chávarri et al.** | 2020 | Evaluation of patient involvement strategies in health technology assessment in Spain: the viewpoint of HTA researchers | Spain | Quantitative survey |
| **Stewart et al.** | 2020 | Scottish Health Technologies Group: enhancing patient engagement | UK | Quantitative survey |
| **Single et al.** | 2021 | Patient advocacy group involvement in health technology assessments: an observational study | South Korea, New Zealand, Taiwan | Quantitative survey |
| **Athanasakis et al.** | 2021 | Giving patients a voice in Health Technology Assessment decision making in Greece: A patient advocacy group consensus analysis | Greece | Qualitative focus group discussion |
| **Scott et al.** | 2017 | Patient advocate perspectives on involvement in HTA: an international snapshot | Canada, UK, The Netherlands, Australia, Taiwan, Japan, Italy, Israel | Quantitative survey |
| **Rocchi et al.** | 2015 | Evolution of health technology assessment: Best practices of the pan Canadian oncology drug review | Canada | Qualitative interviews |
| **Rasburn et al.** | 2020 | Strengthening patient outcome evidence in health technology assessment: a coproduction approach | UK | Quantitative survey, stakeholder dialogue |
| **Pomey et al.** | 2019 | Co construction of health technology assessment recommendations with patients: An example with cardiac defibrillator replacement | Canada | Document analysis, observation research, qualitative interviews |
| **Poder et al.** | 2020 | Patients, users, caregivers, and citizens' involvement in local health technology assessment unit in Quebec: a survey | Canada | Quantitative survey |
| **Elvsaas et al.** | 2020 | Patient involvement in relative effectiveness assessments in the European Network for Health Technology Assessment | Non-country specific | Document analysis |
| **Nabarette et al.** | 2023 | Patient and citizen participation at the organizational level in health technology assessment: An exploratory study in five jurisdictions | Belgium, France, Canada, UK | Quantitative survey, qualitative interviews |
| **Nabarette et al.** | 2018 | Involving patients in the evaluation of health technologies at the French National Authority for Health (HAS) | France | Document analysis, stakeholder discussions |
| **Moran et al.** | 2011 | An uneven spread: A review of public involvement in the National Institute of Health Research's Health Technology Assessment program | UK | Document analysis, qualitative interviews |
| **Messina et al.** | 2012 | A pilot study to identify areas for further improvements in patient and public involvement in health technology assessments for medicines | Australia, Canada, UK | Qualitative interviews |
| **Mercer et al.** | 2020 | Are We Making a Difference? A Qualitative Study of Patient Engagement at the pan Canadian Oncology Drug Review: Perspectives of Patient Groups | Canada | Qualitative interviews |
| **Menon et al.** | 2011 | Role of patient and public participation in health technology assessment and coverage decisions | Australia, Canada, France, Germany, New Zealand, UK, The Netherlands, USA | Literature review |
| **Lopes et al.** | 2020 | The rationale and design of public involvement in health funding decision making: Focus groups with the Canadian public | Canada | Qualitative focus group discussions |
| **Lopes et al.** | 2015 | Involving patients in health technology funding decisions: stakeholder perspectives on processes used in Australia | Australia | Qualitative interviews |
| **Livingstone et al.** | 2020 | Evaluation of the impact of patient input in health technology assessments at NICE | UK | Quantitative survey |
| **Lips et al.** | 2022 | Involvement of Patients and Medical Professionals in the Assessment of Relative Effectiveness: A Need for Closer Cooperation | The Netherlands | Document analysis, qualitative interviews |
| **Kreis et al.** | 2013 | Public engagement in health technology assessment and coverage decisions: a study of experiences in France, Germany, and the United Kingdom | France, Germany, UK | Website review, document analysis, literature review, qualitative interviews |
| **Jommi et al.** | 2018 | Patients' Associations and HTA for medicines: Actual and future role in Italy | Italy | Quantitative survey |
| **Johnson et al.** | 2024 | Exploring Barriers and Facilitators to Patients and Members of the Public Contributing to Rapid Health Technology Assessments for NICE: A Qualitative Study | UK | Qualitative interviews, focus group discussions |
| **Janssens et al.** | 2018 | Patient Involvement in the Lifecycle of Medicines According to Belgian Stakeholders: The Gap Between Theory and Practice | Non-country specific | Qualitative interviews, quantitative survey |
| **Jakab et al.** | 2023 | Recommendations for patient involvement in health technology assessment in Central and Eastern European countries | Bulgaria, Croatia, France, Germany, Greece, Hungary, The Netherlands, Kazakhstan, Lithuania, Poland, North Macedonia, Romania, Serbia, Slovakia, Slovenia, Spain, Turkey, Ukraine, UK | Quantitative survey, qualitative workshop |
| **Holtorf et al.** | 2020 | Pilot approach to analyzing patient and citizen involvement in health technology assessment in four diverse low And middle income countries | South Africa, Brazil, Indonesia, Nigeria | Quantitative survey |
| **Hashem et al.** | 2017 | Decision making in NICE single technological appraisals: How does NICE incorporate patient perspectives? | UK | Document analysis, observation research, qualitative interviews |
| **Hämeen-Anttila et al.** | 2016 | Incorporating patient perspectives in health technology assessments and clinical practice guidelines | Finland | Qualitative focus group discussions |
| **Hailey et al.** | 2013 | Involvement of consumers in health technology assessment activities by inahta agencies | Australia, Austria, Brazil, Canada, Denmark, Finland, France, Germany, Ireland, Israel, Italy, South Korea, Lithuania, Mexico, The Netherlands, Malaysia, Norway, Poland, Spain, Sweden, Taiwan, Thailand, UK | Quantitative survey |
| **Hailey et al.** | 2006 | Survey on the involvement of consumers in health technology assessment programs | Argentina, Australia, Austria, Belgium, Canada, Denmark, France, Germany, Finland, Hungary, Israel, The Netherlands, Mexico, New Zealand, Norway, Spain, Sweden, UK, USA | Quantitative survey |
| **Gunn et al.** | 2023 | A HTA of what? Reframing through including patient perspectives in health technology assessment processes | Ireland, the Netherlands, Norway, UK | Document analysis, qualitative interviews |
| **Gousset et al.** | 2024 | The three domain impact framework for characterizing impact of patient involvement in health technology assessment | UK, Canada, Australia, Brazil, Germany, USA, Spain | Quantitative survey |
| **Georgieva et al.** | 2020 | Role of the Patient's Viewpoint in Health Technologies Assessment in Bulgaria | Bulgaria | Quantitative survey |
| **Gauvin et al.** | 2011 | Moving cautiously: Public involvement and the health technology assessment community | Non-country specific | Qualitative interviews |
| **Gauvin et al.** | 2010 | "It all depends": Conceptualizing public involvement in the context of health technology assessment agencies | Non-country specific | Literature review, website review, qualitative interviews |
| **Abelson et al.** | 2013 | Assessing the impacts of citizen deliberations on the health technology process | Canada | Qualitative focus group discussions |
| **Alcaraz et al.** | 2025 | Design and implementation of a stakeholder consultation process for rapid health technology assessments in Argentina | Argentina | Document analysis |
| **Gagnon et al.** | 2012 | Involving patients in HTA activities at local level: a study protocol based on the collaboration between researchers and knowledge users | Canada | Protocol |
| **Gagnon et al.** | 2011 | Introducing patients' and the public's perspectives to health technology assessment: A systematic review of international experiences | UK, USA, The Netherlands, Canada, Denmark, Germany, New Zealand, Austria | Systematic review |
| **Gagnon et al.** | 2015 | Framework for user involvement in health technology assessment at the local level: views of health managers, user representatives, and clinicians | Canada | Qualitative interviews |
| **Gagnon et al.** | 2012 | Introducing the patient's perspective in hospital health technology assessment (HTA): the views of HTA producers, hospital managers and patients | Canada | Qualitative interviews, focus group discussions |
| **Gagnon et al.** | 2014 | Involving patient in the early stages of health technology assessment (HTA): a study protocol | Canada | Protocol |
| **Facey et al.** | 2010 | Patients' perspectives in health technology assessment: A route to robust evidence and fair deliberation | Non-country specific | Methods |
| **Dipankui et al.** | 2015 | Evaluation of patient involvement in a health technology assessment | Canada | Qualitative interviews |
| **Dipankui et al.** | 2014 | Patient participation in the assessment of alternatives to restraint and seclusion | Canada | Qualitative interviews |
| **Dimitrova et al.** | 2022 | Potential Barriers of Patient Involvement in Health Technology Assessment in Central and Eastern European Countries | Non-country specific | Scoping literature review, qualitative workshop |
| **Desmet et al.** | 2024 | An Inclusive Civil Society Dialogue for Successful Implementation of the EU HTA Regulation: Call to Action to Ensure Appropriate Involvement of Stakeholders and Collaborators | Non-country specific | Qualitative focus group discussions |
| **De Freitas Lopes et al.** | 2020 | Patient and public involvement in health technology decision making processes in Brazil | Brazil | Document analysis |
| **Cook et al.** | 2021 | Development of an international template to support patient submissions in Health Technology Assessments | UK | Quantitative survey, qualitative interviews |
| **Cleemput et al.** | 2020 | Developing an agency's position with respect to patient involvement in health technology assessment: The importance of the organizational culture | Belgium | Qualitative focus group discussion, Delphi panel, interviews, literature review |
| **Cleemput et al.** | 2015 | Acceptability and Perceived Benefits and Risks of Public and Patient Involvement in Health Care Policy: A Delphi Survey in Belgian Stakeholders | Belgium | Quantitative Delphi survey |
| **Chen et al.** | 2022 | Patient Involvement in the Health Technology Assessment Process in Taiwan | Taiwan | Literature review |
| **Castro et al.** | 2018 | Involvement of patients of healthcare systems in health technology assessment (HTA): A narrative review of international strategies | Australia, Germany, Canada, UK, Brazil | Literature review |
| **Boothe et al.** | 2021 | (Re)defining legitimacy in Canadian drug assessment policy? Comparing ideas over time | Canada | Qualitative interviews |
| **Boothe et al.** | 2019 | "Getting to the Table": Changing Ideas about Public and Patient Involvement in Canadian Drug Assessment | Canada | Quantitative survey, qualitative interviews |

*Supplementary materials 4: Mixed Methods Appraisal Tool (MMAT), version 2018, for appraisal of quantitative, qualitative, and mixed-methods studies included in the scoping literature review*

| **Author** | **Year** | **Title** | **MMAT study design** | **MMAT assessment criteria** | | | | |
| --- | --- | --- | --- | --- | --- | --- | --- | --- |
|  |  |  |  | **1** | **2** | **3** | **4** | **5** |
| **Gagnon et al.** | 2021 | Patient and public involvement in health technology assessment: update of a systematic review of international experiences | NA |  |  |  |  |  |
| **Toledo-Chávarri et al.** | 2019 | Toward a Strategy to Involve Patients in Health Technology Assessment in Spain | Qualitative | Y | Y | Y | Y | Y |
| **Kleme et al.** | 2014 | Patient perspective in health technology assessment of pharmaceuticals in Finland | Qualitative | Y | Y | Y | 0 | Y |
| **Abelson et al.** | 2007 | Bringing ‘the public’ into health technology assessment and coverage policy decisions: From principles to practice | NA |  |  |  |  |  |
| **Abelson et al.** | 2016 | Public and patient involvement in health technology assessment: a framework for action | Mixed-methods | Y | N | 0 | Y | N |
| **Atfeh et al.** | 2024 | Involving Patients in Hospital‐Based Health Technology Assessment of Innovative Medical Devices: Adapting to a Specific Local Context and Lessons Learned From the Assessment of an Ex Vivo Perfusion System of Human Donor Hearts | NA |  |  |  |  |  |
| **De Freitas Lopes et al.** | 2023 | Does patient and public involvement impact public health decision making? A 10year retrospective analysis of public consultation in Brazil | Quantitative descriptive | Y | Y | Y | 0 | Y |
| **Toledo-Chávarri et al.** | 2020 | Development of a decisional flowchart for meaningful patient involvement in Health Technology Assessment | NA |  |  |  |  |  |
| **Bidonde et al.** | 2021 | An institutional ethnographic analysis of public and patient engagement activities at a national health technology assessment agency | Mixed-methods | Y | Y | 0 | Y | N |
| **Berglas et al.** | 2016 | Patients' perspectives can be integrated in health technology assessments: an exploratory analysis of CADTH Common Drug Review | Quantitative descriptive | Y | N | Y | NA | Y |
| **Young et al.** | 2017 | Exploring patient and family involvement in the lifecycle of an orphan drug: A scoping review | Qualitative | Y | N | Y | Y | 0 |
| **Wortley et al.** | 2016 | What factors determine the choice of public engagement undertaken by health technology assessment decision making organizations? | NA |  |  |  |  |  |
| **Whitty et al.** | 2013 | An international survey of the public engagement practices of health technology assessment organizations | Quantitative descriptive | Y | N | Y | 0 | Y |
| **Weeks et al.** | 2017 | Evaluation of patient and public involvement initiatives in health technology assessment: a survey of international agencies | Quantitative descriptive | Y | N | Y | 0 | Y |
| **Tran Minh et al.** | 2024 | Experiences of patient organizations' involvement in medicine appraisal and reimbursement processes in Finland: a qualitative study | Qualitative | Y | Y | Y | Y | Y |
| **Toledo-Chávarri et al.** | 2020 | Evaluation of patient involvement strategies in health technology assessment in Spain: the viewpoint of HTA researchers | Quantitative descriptive | Y | Y | Y | Y | N |
| **Stewart et al.** | 2020 | Scottish Health Technologies Group: enhancing patient engagement | Quantitative descriptive | Y | N | Y | 0 | N |
| **Single et al.** | 2021 | Patient advocacy group involvement in health technology assessments: an observational study | Quantitative descriptive | Y | N | Y | 0 | Y |
| **Athanasakis et al.** | 2021 | Giving patients a voice in Health Technology Assessment decision making in Greece: A patient advocacy group consensus analysis | Qualitative | Y | Y | 0 | Y | Y |
| **Scott et al.** | 2017 | Patient advocate perspectives on involvement in HTA: an international snapshot | Quantitative descriptive | Y | N | Y | Y | Y |
| **Rocchi et al.** | 2015 | Evolution of health technology assessment: Best practices of the pan Canadian oncology drug review | Qualitative | Y | Y | Y | Y | Y |
| **Rasburn et al.** | 2020 | Strengthening patient outcome evidence in health technology assessment: a coproduction approach | Mixed-methods | Y | N | 0 | Y | N |
| **Pomey et al.** | 2019 | Co construction of health technology assessment recommendations with patients: An example with cardiac defibrillator replacement | Mixed-methods | Y | Y | Y | Y | N |
| **Poder et al.** | 2020 | Patients, users, caregivers, and citizens' involvement in local health technology assessment unit in Quebec: a survey | Quantitative descriptive | Y | Y | Y | 0 | Y |
| **Elvsaas et al.** | 2020 | Patient involvement in relative effectiveness assessments in the European Network for Health Technology Assessment | Quantitative descriptive | Y | Y | Y | 0 | Y |
| **Nabarette et al.** | 2023 | Patient and citizen participation at the organizational level in health technology assessment: An exploratory study in five jurisdictions | Mixed-methods | Y | Y | 0 | 0 | Y |
| **Nabarette et al.** | 2018 | Involving patients in the evaluation of health technologies at the French National Authority for Health (HAS) | Mixed-methods | Y | Y | 0 | Y | N |
| **Moran et al.** | 2011 | An uneven spread: A review of public involvement in the National Institute of Health Research's Health Technology Assessment program | Mixed-methods | Y | 0 | N | Y | N |
| **Messina et al.** | 2012 | A pilot study to identify areas for further improvements in patient and public involvement in health technology assessments for medicines | Qualitative | Y | Y | Y | Y | Y |
| **Mercer et al.** | 2020 | Are We Making a Difference? A Qualitative Study of Patient Engagement at the pan Canadian Oncology Drug Review: Perspectives of Patient Groups | Qualitative | Y | Y | Y | Y | Y |
| **Menon et al.** | 2011 | Role of patient and public participation in health technology assessment and coverage decisions | NA |  |  |  |  |  |
| **Lopes et al.** | 2020 | The rationale and design of public involvement in health funding decision making: Focus groups with the Canadian public | Qualitative | Y | Y | Y | Y | Y |
| **Lopes et al.** | 2015 | Involving patients in health technology funding decisions: stakeholder perspectives on processes used in Australia | Qualitative | Y | Y | Y | Y | Y |
| **Livingstone et al.** | 2020 | Evaluation of the impact of patient input in health technology assessments at NICE | Mixed-methods | Y | Y | 0 | Y | Y |
| **Lips et al.** | 2022 | Involvement of Patients and Medical Professionals in the Assessment of Relative Effectiveness: A Need for Closer Cooperation | Mixed-methods | Y | Y | Y | Y | Y |
| **Kreis et al.** | 2013 | Public engagement in health technology assessment and coverage decisions: a study of experiences in France, Germany, and the United Kingdom | Mixed-methods | Y | Y | Y | 0 | N |
| **Jommi et al.** | 2018 | Patients' Associations and HTA for medicines: Actual and future role in Italy | Quantitative descriptive | Y | N | Y | N | Y |
| **Johnson et al.** | 2024 | Exploring Barriers and Facilitators to Patients and Members of the Public Contributing to Rapid Health Technology Assessments for NICE: A Qualitative Study | Qualitative | Y | Y | Y | Y | Y |
| **Janssens et al.** | 2018 | Patient Involvement in the Lifecycle of Medicines According to Belgian Stakeholders: The Gap Between Theory and Practice | Mixed-methods | Y | N | Y | Y | Y |
| **Jakab et al.** | 2023 | Recommendations for patient involvement in health technology assessment in Central and Eastern European countries | Mixed-methods | Y | N | Y | Y | Y |
| **Holtorf et al.** | 2020 | Pilot approach to analyzing patient and citizen involvement in health technology assessment in four diverse low And middle income countries | Quantitative descriptive | Y | N | Y | 0 | Y |
| **Hashem et al.** | 2017 | Decision making in NICE single technological appraisals: How does NICE incorporate patient perspectives? | Mixed-methods | Y | Y | 0 | Y | Y |
| **Hämeen-Anttila et al.** | 2016 | Incorporating patient perspectives in health technology assessments and clinical practice guidelines | Qualitative | Y | Y | Y | Y | Y |
| **Hailey et al.** | 2013 | Involvement of consumers in health technology assessment activities by inahta agencies | Quantitative descriptive | Y | Y | Y | 0 | Y |
| **Hailey et al.** | 2006 | Survey on the involvement of consumers in health technology assessment programs | Quantitative descriptive | Y | Y | Y | 0 | Y |
| **Gunn et al.** | 2023 | A HTA of what? Reframing through including patient perspectives in health technology assessment processes | Mixed-methods | Y | Y | 0 | Y | Y |
| **Gousset et al.** | 2024 | The three domain impact framework for characterizing impact of patient involvement in health technology assessment | Quantitative descriptive | 0 | N | Y | 0 | Y |
| **Georgieva et al.** | 2020 | Role of the Patient's Viewpoint in Health Technologies Assessment in Bulgaria | Quantitative descriptive | Y | N | Y | 0 | Y |
| **Gauvin et al.** | 2011 | Moving cautiously: Public involvement and the health technology assessment community | Qualitative | Y | Y | Y | Y | Y |
| **Gauvin et al.** | 2010 | "It all depends": Conceptualizing public involvement in the context of health technology assessment agencies | Mixed-methods | Y | Y | Y | Y | N |
| **Abelson et al.** | 2013 | Assessing the impacts of citizen deliberations on the health technology process | Qualitative | Y | 0 | Y | Y | Y |
| **Alcaraz et al.** | 2025 | Design and implementation of a stakeholder consultation process for rapid health technology assessments in Argentina | Quantitative descriptive | Y | Y | Y | NA | Y |
| **Gagnon et al.** | 2012 | Involving patients in HTA activities at local level: a study protocol based on the collaboration between researchers and knowledge users | NA |  |  |  |  |  |
| **Gagnon et al.** | 2011 | Introducing patients' and the public's perspectives to health technology assessment: A systematic review of international experiences | NA |  |  |  |  |  |
| **Gagnon et al.** | 2015 | Framework for user involvement in health technology assessment at the local level: views of health managers, user representatives, and clinicians | Qualitative | Y | Y | Y | Y | Y |
| **Gagnon et al.** | 2012 | Introducing the patient's perspective in hospital health technology assessment (HTA): the views of HTA producers, hospital managers and patients | Qualitative | Y | Y | Y | Y | Y |
| **Gagnon et al.** | 2014 | Involving patient in the early stages of health technology assessment (HTA): a study protocol | NA |  |  |  |  |  |
| **Facey et al.** | 2010 | Patients' perspectives in health technology assessment: A route to robust evidence and fair deliberation | NA |  |  |  |  |  |
| **Dipankui et al.** | 2015 | Evaluation of patient involvement in a health technology assessment | Qualitative | Y | Y | Y | Y | Y |
| **Dipankui et al.** | 2014 | Patient participation in the assessment of alternatives to restraint and seclusion | Qualitative | Y | Y | Y | Y | Y |
| **Dimitrova et al.** | 2022 | Potential Barriers of Patient Involvement in Health Technology Assessment in Central and Eastern European Countries | Qualitative | Y | Y | Y | Y | Y |
| **Desmet et al.** | 2024 | An Inclusive Civil Society Dialogue for Successful Implementation of the EU HTA Regulation: Call to Action to Ensure Appropriate Involvement of Stakeholders and Collaborators | Qualitative | Y | Y | Y | Y | Y |
| **De Freitas Lopes et al.** | 2020 | Patient and public involvement in health technology decision making processes in Brazil | Quantitative descriptive | Y | Y | Y | NA | Y |
| **Cook et al.** | 2021 | Development of an international template to support patient submissions in Health Technology Assessments | Mixed-methods | Y | N | N | Y | N |
| **Cleemput et al.** | 2020 | Developing an agency's position with respect to patient involvement in health technology assessment: The importance of the organizational culture | Qualitative | Y | Y | Y | Y | Y |
| **Cleemput et al.** | 2015 | Acceptability and Perceived Benefits and Risks of Public and Patient Involvement in Health Care Policy: A Delphi Survey in Belgian Stakeholders | Quantitative descriptive | Y | N | Y | 0 | Y |
| **Chen et al.** | 2022 | Patient Involvement in the Health Technology Assessment Process in Taiwan | NA |  |  |  |  |  |
| **Castro et al.** | 2018 | Involvement of patients of healthcare systems in health technology assessment (HTA): A narrative review of international strategies | NA |  |  |  |  |  |
| **Boothe et al.** | 2021 | (Re)defining legitimacy in Canadian drug assessment policy? Comparing ideas over time | Qualitative | Y | Y | Y | Y | 0 |
| **Boothe et al.** | 2019 | "Getting to the Table": Changing Ideas about Public and Patient Involvement in Canadian Drug Assessment | Mixed-methods | Y | Y | Y | Y | Y |

*NA: Not applicable, Y: Yes, N: No, 0: cannot tell*

*The Mixed Methods Appraisal Tool (MMAT) was used as critical appraisal tool to appraise relevant study designs, including the empirical studies (quantitative, qualitative, and mixed-methods study designs). Literature reviews were not appraised, as MMAT is not designed for these study types.*

Hong QN, Pluye P, Fàbregues S, Bartlett G, Boardman F, Cargo M, Dagenais P, Gagnon M-P, Griffiths F, Nicolau B, O’Cathain A, Rousseau M-C, Vedel I. Mixed Methods Appraisal Tool (MMAT), version 2018. Registration of Copyright (#1148552), Canadian Intellectual Property Office, Industry Canada.
